# A Bayesian network meta‐analysis of ALK inhibitor treatments in patients with ALK‐positive non‐small cell lung cancer

**DOI:** 10.1002/cam4.6241

**Published:** 2023-06-19

**Authors:** Bei Zheng, Hong Jiang, Wenjuan Yang, Ying Li, Bingqing Liang, Jun Zhu, Nanmei Chen, Miao Chen, Meiling Zhang

**Affiliations:** ^1^ Department of Pharmacy Tongde Hospital Zhejiang Province Hangzhou China; ^2^ Department of Pharmacy Zhejiang Academy of Traditional Chinese Medicine Hangzhou China

**Keywords:** ALK inhibitor, Bayesian, network meta‐analysis, non‐small cell lung cancer

## Abstract

**Objective:**

To date, no direct comparisons have compared the effectiveness of all ALK inhibitors (ALKis) against ALK‐positive non‐small cell lung cancer (NSCLC). The aim of the present study was to investigate the efficacy and safety of ALKis in ALK‐positive NSCLC.

**Methods:**

The effectiveness of ALKis was evaluated by assessing progression‐free survival (PFS), overall survival (OS), overall response rate (ORR), and PFS with baseline brain metastasis (BM). The serious adverse events (SAEs: Grade ≥ 3) and adverse events (AEs) leading to discontinuation were pooled to evaluate safety. We conducted an indirect treatment comparison between all ALKis by using a Bayesian model.

**Results:**

Twelve eligible trials including seven treatments were identified. All of the ALKis improved PFS and ORR relative to chemotherapy. Consistent with alectinib, brigatinib, lorlatinib, and ensartinib showed significant differences versus crizotinib and ceritinib. Lorlatinib seemed to prolong PFS compared with alectinib (0.64, 0.37 to 1.07), brigatinib (0.56, 0.3 to 1.05), and ensartinib (0.53, 0.28 to 1.02). No significant difference was found among them in OS except for alectinib versus crizotinib. Moreover, alectinib was significantly more effective than crizotinib (1.54, 1.02 to 2.5) in achieving the best ORR. Subgroup analyses based on BM indicated that PFS was dramatically lengthened by lorlatinib. Compared with other ALKis, alectinib notably reduced the rate of SAEs. There was no striking difference between discontinuation for AEs, except for ceritinib versus crizotinib. The ranking of validity showed that lorlatinib had the longest PFS (98.32%) and PFS with BM (85.84%) and the highest ORR (77.01%). The rank of probabilities showed that alectinib had the potentially best safety in terms of SAEs (97.85%), and ceritinib had less discontinuation (95.45%).

**Conclusion:**

Alectinib was the first choice for patients with ALK‐positive NSCLC and even for those with BM, whereas lorlatinib was the second choice. Long‐term follow‐up and prospective studies are warranted to compare ALKis and to verify our conclusions directly.

## 1 INTRODUCTION

Anaplastic lymphoma kinase (ALK) gene rearrangements are oncogenic drivers^1^ in nearly 2%–7% of non‐small cell lung cancer (NSCLC) cases.^2^ Furthermore, the echinoderm microtubule‐associated protein‐like 4‐ALK (EML4‐ALK) gene plays a key role in tumorigenesis in circa 5% of NSCLC.^3^

Compared with conventional chemotherapeutic regimens, ALK inhibitors (ALKis) have exceptional therapeutic efficacy in ALK‐positive NSCLC. To date, six ALKis (crizotinib, alectinib, ceritinib, brigatinib, lorlatinib, and ensartinib) have been extensively used in clinical settings. Crizotinib, as the first generation ALKi, has demonstrated superior antineoplastic activity and safety to chemotherapy in a series of clinical trials (PROFILE1007 and PROFILE1014).^4^, ^5^ Nevertheless, frequent crizotinib resistance and poor central nervous system concentration have been challenges for oncologists.^6^ Second‐generation ALKis (brigatinib and alectinib) and third‐generation ALKis (lorlatinib and ensartinib) can provide effective treatment options.^7^ In addition, more effective agents are being investigated in ongoing clinical studies, such as entrectinib and belizatinib.^8^ New agents have been considered crucial breakthroughs in treating ALK‐positive NSCLC, which has led to the dilemma of optimal therapy options..

It is challenging to treat brain metastases (BM) in NSCLC patients due to the low treatment exposure and response rates in the central nervous system (CNS). Chemotherapy and crizotinib have limited intracranial penetration,^9^ which demonstrates poor efficacy in avoiding intracranial progression.

Subsequently, alectinib and ceritinib have shown the ability to cross the blood–brain barrier.^10^ Second‐generation ALKis prolonged progression‐free survival (PFS), but drug resistance and intracranial tumour relapse were subsequently reported.^11^ It is promising that third‐generation ALKis displayed a better intracranial response to crizotinib.^12^ To date, the intracranial effectiveness discrepancy between second‐ and third‐generation ALKis has rarely been examined, and minimal evidence is available between them in ALK‐positive NSCLC with BM. Previously published network meta‐analyses (NMAs) did not include ensartinib and have not yet examined all of the marketed ALKis. Furthermore, new data for several ALKis have been updated (ALTA‐1 L, CROWN, and eXalt3 studies).^13^, ^14^, ^15^ Overall, alectinib, brigatinib, or lorlatinib have become the first‐line regimens, whereas ceritinib is the “other recommended” option, and crizotinib is recommended as “useful in certain circumstances” in the NCCN guidelines (2022 v3) for ALK‐positive NSCLC.

The relative effectiveness and toxicity of all these ALKis were limited by the lack of sufficient data from comparison trials. Thus, updates are necessary. Nevertheless, the first treatment for ALK‐positive NSCLC remains contentious. Consequently, we conducted a Bayesian NMA to synthetically evaluate the effectiveness and safety of ALKis on ALK‐positive NSCLC, even for those with BM. We hoped to overcome the limitations of sample size and face‐to‐face comparison through our study to conclude the effectiveness ranking and provide theoretical recommendations for clinical practice.

## 2 MATERIALS AND METHODS

### 2.1 Search strategy

Our study was executed according to the Preferred Reporting Items for the PRISMA extension statement for NMA.^16^ We searched the Cochrane Library, PubMed, ClinicalTrials, Embase, and Medline to identify relevant studies using the main search terms “NSCLC” and “ALK inhibitors” for randomized controlled trials (RCTs) until the end of June 2022 without language or date restrictions. Subsequently, abstracts and presentations of ongoing RCTs on NSCLC were checked from several of the most important international conferences from 2014 to 2022. Finally, the additional articles were subjoined by inspecting the references of the relevant articles. The search strategies have been shown in the Supporting Information Appendix 1.

### 2.2 Inclusion and exclusion criteria (predefined PICOS)

The potentially eligible studies were extracted by two authors, and any disagreements were resolved via consensus.

Population: Treatment‐naïve or experienced participants with phase III or IV ALK‐positive NSCLC.

*Interventions*: ALKis.

*Comparators*: Placebo, chemotherapy, another ALKis.

*Outcomes*: The primary outcome was PFS. The secondary outcomes were overall survival (OS), overall response rate (ORR), PFS with BM, serious adverse events (SAEs), and adverse events (AEs) leading to discontinuation.

Study design: RCTs.

### 2.3 Data extraction and risk of bias

Data were extracted independently by two authors, and all disagreements were resolved by consensus. The following data were extracted: study characteristics (e.g., trial name, first author, publication date, design), participant characteristics (e.g., age, sex, number, treatment history, smoking status), cancer characteristics (e.g., histopathology, stage, BM, performance status), antitumor efficacy (e.g., PFS, OS, ORR, PFS with BM), and number of AEs (SAEs: grade ≥ 3, treatment discontinuation). Where PFS was assessed by both study investigators as well as by independent review committees, we extracted and analyzed data from the latter.

Risk of bias (ROB) was independently evaluated by two authors utilizing the Cochrane Collaboration's ROB tool for RCTs.^17^ Specifically, we assigned a judgment of high, low, or unclear ROB for the domains of random sequence generation, allocation concealment, blinding of participants and personnel, blinding of outcome assessments, incomplete outcome data, selective outcome reporting, and other sources of bias (Appendix 2).

### 2.4 Data synthesis and statistical analysis

PFS was defined as the time from the date of randomization to the date of the first documentation of objective tumour progression or death on study due to any cause, whichever occurred first. OS was defined as the time from randomization to the date of death due to any cause. ORR was defined as the percentage of patients with complete response (CR) or partial response (PR). SAEs were defined as an adverse event that results in death, is life‐threatening, requires inpatient hospitalization or extends a current hospital stay, results in ongoing or significant incapacity or interferes substantially with normal life functions, or causes a congenital anomaly or birth defect. AEs leading to discontinuation were defined as the number of patients in which treatment was discontinued due to intolerance of adverse events. The effectiveness and safety of direct and indirect evidence were synthesized in ALKis. The survival outcomes (PFS, OS, PFS with BM) were reported as hazard ratios, and binary outcomes (ORR, SAEs, and AEs leading to discontinuation) were reported as odds ratios, along with corresponding 95% credible intervals (CI).

We generated network plots to clarify whether directly or indirectly. We executed a pairwise meta‐analysis on face‐to‐face comparisons to evaluate heterogeneity by employing the *Q* test and *I* ^2^ statistic within a visual forest plot. Heterogeneity was considered to be high for estimated *I* ^2^ values over 50%.^18^ Comparisons between random effects (RE) and fixed effects (FE) model estimates were performed to evaluate whether the additional variability was affecting the NMA results in terms of estimates and related variability. Hence, heterogeneity was also evaluated by comparing RE and FE model estimates via a graphical method, specifically with the use of a Bland–Altman plot.^19^

The NMA was executed in a Bayesian framework utilizing a Markov chain Monte Carlo simulation technique. The random effects model was applied, which accounted for between‐trial heterogeneity.^20^ The model used four chains with overdispersed initial values, with Gibbs sampling based on 50,000 iterations after a burn‐in phase of 10,000 iterations with a thinning factor of 2.5. The Brooks‐Gelman‐Rubin diagnostic tool and visual inspections of time series and density plots (Supporting Information Figure S1) were used to evaluate the convergence. Within the Bayesian framework, the surface under the cumulative ranking curve (SUCRA) was applied with a rankogram plot.^21^ The hierarchy of treatments was provided with the consideration of both the location and the variance of all relative treatment effects.

We assessed global inconsistencies for the deviance information criterion in consistency and inconsistency models.^22^ The local inconsistencies were evaluated by comparing the traditional pairwise meta‐analyses in the frequentist framework and NMA in the Bayesian framework. The inconsistency of the model was calculated by the node‐splitting approach. In addition, our study evaluated publication biases and small‐study effects via visual inspections of comparison‐adjusted and contour‐enhanced funnel plots, complemented by Egger and Begg's tests. The trim and fill method was also used to analyze the cause of publication bias.

To evaluate the robustness and reliability of the results, we carried out various sensitivity analyses. Subgroup analyses were also conducted in the initial sensitivity analyses according to whether the patients had BM. Second, we erased every trial from the other trials when the results indicated a mean effect that could potentially be driven by individual studies. The third analysis omitted the PROFILE 1029 study to check the sources of heterogeneity. All analyses were executed using R (v.4.1.3) software.

## 3 RESULTS

### 3.1 Identification of studies and study quality

**FIGURE 1:**
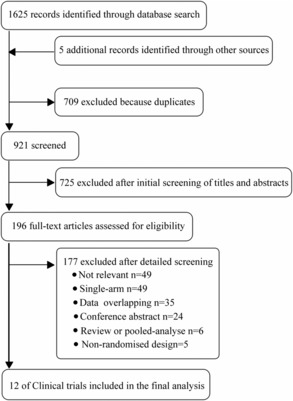
Study selection.

**FIGURE 2:**
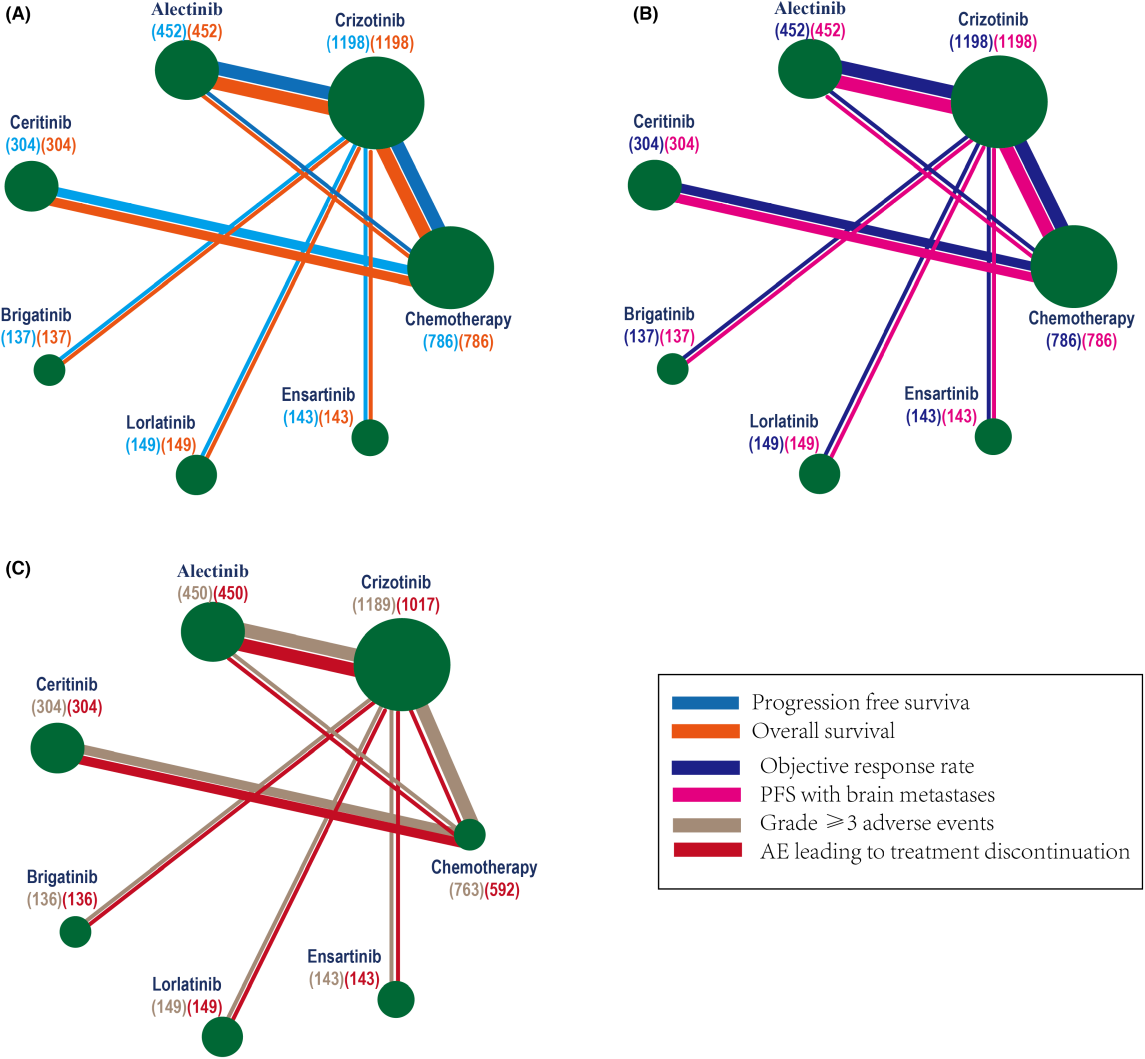
Network diagrams of the comparisons of treatments in different groups of patients with ALK‐positive NSCLC. (A) PFS and OS. (B) ORR and PFS with BM. (C) AEs of grade 3 or higher and AEs leading to treatment discontinuation.

A total of 12 trials (*n* = 3169) and seven treatments (crizotinib, alectinib, brigatinib, lorlatinib, ensartinib, and chemotherapy) were included. The screening process and the network are shown in Figures 1 and 2. We used data from RCTs^4^, ^5^, ^13^, ^14^, ^15^, ^23^, ^24^, ^25^, ^26^, ^27^, ^28^, ^29^ with longer follow‐up times than have been previously published. The key characteristics and clinical outcomes are described in Tables 1 and 2. Most of the trials were open‐label, which potentially induced performance biases (Appendix 2). Nevertheless, knowledge of group assignments would not be expected to affect objective outcome assessments such as OS, PFS, ORR, and SAEs.

**TABLE 1:** Patient characteristics and disease‐related demographics of RCTs.

Author, yr, page (study name; NCT no.)	Trial phase	Treatment	Number of patients	Age, yr, median (range)	Male (%)	Current smoking (%)	Never smoked (%)	Brain metastases (%)	ECOG0‐1 (%)	ECOG2 (%)	Adenocarcinoma	Crossover
Nakagawa^13^ (J‐ALEX; JapicCTI‐132,316; JO28928)	III	Alectinib 300 mg BID	103	61 (27–85)	39.81	1.94	54.37	13.59	98.06	1.94	94.17%	No
		Crizotinib 250 mg BID	104	59.5 (25–84)	39.42	2.88	58.65	0.28	98.08	1.92	95.19%	
Ross Camidge^23^ (ALTA‐1 L Study, NCT02737501)	III	Brigatinib 180 mg QD	137	58 (27–86)	49.64	2.92	61.31	29.20	94.89	4.38	91.97%	Yes
		Crizotinib 250 mg BID	138	60 (29–89)	41.30	5.07	54.35	29.71	94.93	4.35	99.28%	
Shaw^14^ (CROWN NCT03052608)	III	Lorlatinib 100 mg QD	149	61 (51–69)	43.62	45.64	54.36	25.50	97.99	2.01	93.96%	No
		Crizotinib 250 mg BID	147	56 (45–66)	38.10	35.37	63.95	27.21	93.88	6.12	95.24%	
Horn^15^ eXalt3 NCT02767804	III	Ensartinib 225 mg QD	143	54 (25–86)	50.30	40.60	59.40	32.90	95.10	4.90	/	No
		Crizotinib 250 mg BID	147	53 (26–90)	52.40	36.10	63.90	38.80	95.20	4.80	/	
Mok^24^ (ALEX, NCT02075840)	III	Alectinib 600 mg BID	152	58(25–88)	44.74	7.89	60.53	42.11	93.42	6.58	90.13%	No
		Crizotinib 250 mg BID	151	54 (18–91)	42.38	3.31	64.90	38.41	93.38	6.62	94.04%	
Zhou^25^ (ALESIA study, NCT02838420)	III	Alectinib 600 mg BID	125	51 (43–59)	51.20	3.20	67.20	35.20	96.80	3.20	93.60%	No
		Crizotinib 250 mg BID	62	49 (41–59)	54.84	4.84	72.58	37.10	98.39	1.61	95.16%	
Wu^26^ (PROFILE 1029, NCT01639001)	III	Crizotinib 250 mg BID	104	48 (24–67)	48.08	6.73	75.00	20.19	96.15	3.85	96.15%	Not reported
		Standard platinum‐based chemotherapy	103	50 (23–69)	41.75	8.74	69.90	31.07	96.12	3.88	98.06%	
Solomon^4^ (PROFILE 1014, NCT01154140)	III	Crizotinib 250 mg BID	172	52 (22–76)	39.53	5.81	61.63	26.16	93.60	6.00	93.60%	Yes
		Standard platinum‐based chemotherapy	171	54 (19–78)	36.84	2.92	65.50	27.49	95.32	5.00	94.15%	
Nishio^5^ (PROFILE 1007, NCT00932893)	III	Crizotinib 250 mg BID	173	51 (22–81)	43.35	2.89	62.43	34.68	87.28	10.98	94.22%	No
		Standard platinum‐based chemotherapy	174	49 (24–85)	44.83	5.17	63.79	34.48	91.38	8.62	91.95%	
Novello^27^ (ALUR, NCT02604342)	III	Alectinib 600 mg BID	72	55.5 (21–82)	56.94	2.78	48.61	38.89	91.67	8.33	100.00%	Yes
		Standard platinum‐based chemotherapy	35	59 (37–80)	48.57	5.71	45.71	42.86	85.71	14.29	100.00%	
Soria^28^ (ASCEND‐4, NCT01828099)	III	Ceritinib 750 mg QD	189	55 (22–81)	46.03	7.94	57.14	31.22	93.12	6.88	95.24%	Yes
		Standard platinum‐based chemotherapy	187	54 (22–80)	39.04	8.02	65.24	33.16	93.58	5.88	97.86%	
Shaw^29^ (ASCEND‐5, NCT01828112)	III	Ceritinib 750 mg QD	115	54 (44–63)	40.87	3.48	61.74	56.52	92.17	7.83	96.52%	Yes
		Standard platinum‐based chemotherapy	116	54 (47–64)	47.41	0.86	52.59	59.48	95.69	4.31	97.41%	

**TABLE 2:** General characteristics of clinical trials.

Author, yr (study; NCT no.)	Treatment	Number of patients	PFS HR (95% CI)	OS HR (95% CI)	ORR (%)	PFS‐BM HR (95% CI)	Grade ≥ 3 AEs (%)	AEs leading to treatment discontinuation (%)
Nakagawa^13^ (J‐ALEX, JapicCTI‐132,316, JO28928)	Alectinib 300 mg BID	103	0.37 (0.26–0.52)	0.80 (0.35–1.82)	92.23	0.08 (0.01–0.61)	36.89	11.65%
	Crizotinib 250 mg BID	104			78.85		60.58	23.08%
Ross Camidge^23^ (ALTA‐1LStudy, NCT02737501)	Brigatinib 90 mg QD for 7 days, then 180 mg QD	137	0.48 (0.35–0.66)	0.54 (0.31–0.92)	74.45	0.293 (0.17–0.51)	69.85	13.24%
	Crizotinib 250 mg BID	138			62.32		56.20	8.76%
Shaw^14^ (CROWN NCT03052608)	Lorlatinib 100 mg QD	149	0.27 (0.184–0.388)	0.72 (0.57–1.47)	77.18	0.21 (0.099–0.436)	64.43	63.76%
	Crizotinib 250 mg BID	147			58.50		38.03	58.45%
Horn^15^ (eXalt3, NCT02767804)	Ensartinib 225 mg QD	143	0.51 (0.35–0.72)	0.91 (0.54–1.54)	74.13	0.55 (0.3–1.01)	50.35	9.09%
	Crizotinib 250 mg BID	147			66.67		42.47	6.85%
Mok^24^ (ALEX, NCT02075840)	Alectinib 600 mg BID	152	0.43 (0.32–0.58)	0.67 (0.46–0.98)	82.89	0.37 (0.23–0.58)	51.97	14.47%
	Crizotinib 250 mg BID	151			75.50		56.29	14.57%
Zhou^25^ (ALESIA study, NCT02838420)	Alectinib 600 mg BID	125	0.37 (0.22–0.61)	0.28 (0.12–0.68)	91.20	0.11 (0.05–0.28)	28.80	7.20%
	Crizotinib 250 mg BID	62			77.42		48.39	9.68%
Wu^26^ (PROFILE 1029, NCT01639001)	Crizotinib 250 mg BID	104	0.402 (0.286–0.565)	1.056 (0.734–1.521)	87.50	0.507 (0.258–0.994)	41.35	8.65%
	Standard platinum‐based chemotherapy	103			45.63		67.33	1.98%
Solomon^4^ (PROFILE 1014, NCT01154140)	Crizotinib 250 mg BID	172	0.45 (0.35–0.60)	0.760 (0.548–1.053)	74.42	0.4 (0.23–0.69)	50.29	15.79%
	Standard platinum‐based chemotherapy	171			45.03		53.25	9.47%
Nishio^5^ (PROFILE 1007, NCT00932893)	Crizotinib 250 mg BID	173	0.487 (0.371–0.638)	0.854 (0.661–1.104)	65.32	0.67 (0.44–1.03)	25.58	/
	Standard platinum‐based chemotherapy	174			19.54		24.56	/
Novello^27^ (ALUR, NCT02604342;)	Alectinib 600 mg BID	72	0.32 (0.17–0.59)	0.89 (0.35–2.24)	37.50	0.12 (0.05–0.27)	27.14	5.71%
	Standard platinum‐based chemotherapy	35			2.86		41.18	8.82%
Soria^28^ (ASCEND‐4, NCT01828099)	Ceritinib 750 mg QD	189	0.55 (0.42–0.73)	0.73 (0.50–1.08)	72.49	0.7 (0.44–1.12)	78.31	5.29%
	Standard platinum‐based chemotherapy	187			26.74		61.71	11.43%
Shaw^29^ (ASCEND‐5, NCT01828112)	Ceritinib 750 mg QD	115	0.49 (0.36–0.67)	1.0 (0.67–1.49)	39.13	0.54 (0.36–0.8)	90.43	3.48%
	Standard platinum‐based chemotherapy	116			6.90		80.53	5.31%

### 3.2 Bayesian network meta‐analyses

#### 3.2.1 Progression‐free survival

Chemotherapy was the worst of the ALKis. All of the ALKis substantially prolonged PFS compared with chemotherapy (Table 3A). Furthermore, alectinib (hazard ratio [HR]: 0.34, 95% credible interval [95% CI]: 0.16 to 0.71) had an advantage over ceritinib. Alectinib was also superior to crizotinib (HR: 0.42, 95% CI: 0.33 to 0.54) in providing the best PFS benefit, and substantial variation was also observed in other ALKis (brigatinib, lorlatinib, and ensartinib). Pooled HRs also indicated that alectinib‐treated patients had the advantage of PFS over brigatinib or ensartinib‐treated patients. Moreover, lorlatinib had a longer PFS than ensartinib or alectinib. Nevertheless, there was no significant difference among alectinib, brigatinib, lorlatinib, and ensartinib.

**TABLE 3:** Pooled estimates of the NMA. (A) PFS and OS. (B) ORR and PFS with BM. (C) AEs of grade ≥3 and AEs leading to treatment discontinuation.

A. Progression‐free survival (PFS) and overall survival (OS)						
Crizotinib	**0.42 (0.33, 0.54)**	1.11 (0.76, 1.57)	**0.48 (0.32, 0.73)**	**0.27 (0.17, 0.43)**	**0.5 (0.32, 0.79)**	**2.12 (1.68, 2.61)**
**1.57 (1.04, 2.42)**	Alectinib	**2.64 (1.7, 3.98)**	1.14 (0.69, 1.85)	0.64 (0.37, 1.07)	1.19 (0.71, 1.99)	**5.03 (3.64, 6.8)**
1.05 (0.62, 1.88)	0.67 (0.35, 1.31)	Ceritinib	**0.43 (0.25, 0.76)**	**0.24 (0.14, 0.44)**	**0.45 (0.26, 0.82)**	**1.9 (1.44, 2.54)**
1.86 (0.89, 3.9)	1.19 (0.5, 2.76)	1.77 (0.69, 4.37)	Brigatinib	0.56 (0.3, 1.05)	1.04 (0.56, 1.93)	**4.41 (2.71, 6.99)**
1.1 (0.55, 2.19)	0.7 (0.31, 1.53)	1.05 (0.42, 2.46)	0.59 (0.21, 1.61)	Lorlatinib	1.87 (0.98, 3.59)	**7.9 (4.69, 13.13)**
1.1 (0.53, 2.26)	0.7 (0.3, 1.59)	1.05 (0.41, 2.54)	0.59 (0.21, 1.66)	1.01 (0.37, 2.69)	Ensartinib	**4.23 (2.53, 6.94)**
0.89 (0.67, 1.3)	**0.57 (0.35, 0.95)**	0.85 (0.55, 1.34)	0.48 (0.22, 1.12)	0.81 (0.39, 1.8)	0.81 (0.38, 1.85)	Chemotherapy

B. Objective response rate (ORR) and PFS with BM						
Crizotinib	**1.54 (1.02, 2.5)**	1.4 (0.65, 3.03)	1.4 (0.63, 3.12)	1.68 (0.76, 3.73)	1.23 (0.55, 2.7)	**0.3 (0.18, 0.45)**
**4.34 (2.47, 9.5)**	Alectinib	0.91 (0.37, 2.09)	0.91 (0.35, 2.19)	1.09 (0.42, 2.61)	0.8 (0.31, 1.89)	**0.19 (0.1, 0.33)**
0.87 (0.35, 2.18)	**0.2 (0.06, 0.51)**	Ceritinib	1 (0.33, 3)	1.2 (0.4, 3.57)	0.87 (0.29, 2.61)	**0.21 (0.11, 0.38)**
**3.41 (1.17, 9.86)**	0.79 (0.19, 2.43)	3.92 (0.95,16.04)	Brigatinib	1.2 (0.39, 3.66)	0.88 (0.28, 2.66)	**0.21 (0.08, 0.51)**
**4.83 (1.51, 15.55)**	1.11 (0.26, 3.85)	**5.57 (1.28,24.52)**	1.42 (0.29, 7.02)	Lorlatinib	0.73 (0.24, 2.23)	**0.18 (0.07, 0.42)**
1.81 (0.61, 5.45)	0.42 (0.1, 1.34)	2.08 (0.5, 8.65)	0.53 (0.12, 2.49)	0.38 (0.08, 1.88)	Ensartinib	**0.24 (0.09, 0.58)**
0.53 (0.3, 0.94)	**0.12 (0.05, 0.24)**	0.61 (0.3, 1.25)	**0.16 (0.05, 0.53)**	**0.11 (0.03, 0.4)**	0.29 (0.09, 1)	Chemotherapy

C. AEs of grade ≥ 3 and AEs leading to discontinuation						
Crizotinib	**0.64 (0.42, 0.98)**	1.93 (0.96, 3.79)	1.45 (0.67, 3.13)	2.14 (0.98, 4.68)	1.26 (0.58, 2.75)	1.26 (0.83, 1.91)
1.63 (0.89, 3.34)	Alectinib	**2.98 (1.4, 6.39)**	2.25 (0.95, 5.45)	**3.32 (1.39, 8.15)**	1.96 (0.82, 4.78)	**1.96 (1.16, 3.36)**
**3.68 (1.06, 12.72)**	2.24 (0.57, 8.2)	Ceritinib	0.75 (0.27, 2.1)	1.11 (0.4, 3.14)	0.65 (0.23, 1.87)	0.66 (0.38, 1.13)
0.69 (0.21, 2.27)	0.42 (0.1, 1.57)	0.19 (0.03, 1.05)	Brigatinib	1.47 (0.49, 4.39)	0.87 (0.29, 2.57)	0.87 (0.36, 2.08)
0.87 (0.31, 2.46)	0.53 (0.14, 1.71)	0.24 (0.05, 1.16)	1.24 (0.26, 6.15)	Lorlatinib	0.59 (0.19, 1.77)	0.59 (0.24, 1.43)
0.82 (0.23, 2.85)	0.5 (0.11, 1.97)	0.22 (0.04, 1.3)	1.17 (0.21, 6.76)	0.94 (0.19, 4.76)	Ensartinib	1 (0.41, 2.43)
1.68 (0.75, 3.78)	1.03 (0.38, 2.54)	0.46 (0.18, 1.18)	2.41 (0.58, 10.5)	1.93 (0.53, 7.3)	2.07 (0.46, 9.22)	Chemotherapy

*Note*: The column treatment was compared with the row treatment. Numbers in parentheses are the 95% CI. ORs or HRs with a Bayesian *p*‐value of less than 0.05 are in bold. (A) PFS use the white colour and OS use the faint yellow. (B) ORR use the white colour and PFS with BM use the faint yellow. (C) AEs of grade ≥3 use the white colour and AEs leading to discontinuation use the faint yellow.

#### 3.2.2 Overall survival

In terms of OS (Table 3A), alectinib (HR: 1.57, 95% CI: 1.04 to 2.42) not only had a better benefit than crizotinib but also had significant benefits versus chemotherapy (HR: 0.57, 95% CI: 0.35 to 0.95). Alectinib had advantages over ceritinib, lorlatinib, and ensartinib. Moreover, brigatinib had a longer OS than ceritinib, lorlatinib, and ensartinib. However, a statistically significant difference was not observed. Similar efficacy was found between alectinib and brigatinib and among ceritinib, crizotinib, lorlatinib, and ensartinib.

#### 3.2.3 Objective response rate

Chemotherapy significantly decreased the ORR compared with ALKis (Table 3B), which was shown to have the worst ORR. Alectinib produced a significant ORR advantage over crizotinib (HR: 1.54, 95% CI: 1.02 to 2.5). Nevertheless, a significant difference was not verified in other analogous ALKis.

#### 3.2.4 PFS with BM

Lorlatinib and alectinib displayed consistent statistical PFS superiority in ALK‐positive NSCLC with BM (Table 3B). Additionally, compared with crizotinib or ceritinib, meaningful discrepancies in effectiveness were demonstrated in lorlatinib and alectinib. In terms of PFS with BM, brigatinib (HR: 3.41, 95% CI: 1.17 to 9.86) provided a PFS benefit over crizotinib. The significant differences in lorlatinib versus chemotherapy (HR: 0.11, 95% CI: 0.03 to 0.4), alectinib versus chemotherapy (HR: 0.12, 95% CI: 0.05 to 0.24), and brigatinib versus chemotherapy (HR: 0.16, 95% CI: 0.05 to 0.53) were in favor of lorlatinib as the best option among the comparable treatments. Furthermore, the marginal difference in ensartinib was slightly inferior to chemotherapy (HR: 0.29, 95% CI: 0.09 to 1).

#### 3.2.5 Toxic effects

Under clinical recommendations, AEs should be considered and potentially avoided. We summarized the comparisons of SAEs (grade ≥ 3) and AEs leading to discontinuation (Table 3C). Lorlatinib had the highest risk of SAEs, whereas alectinib was the safest. Both ALKis versus chemotherapy and second/third‐generation ALKis versus crizotinib had no significant difference in SAEs, except for alectinib versus crizotinib or ceritinib or chemotherapy. Furthermore, there was no difference with respect to AEs leading to discontinuation, except for the fact that ceritinib had an advantage over crizotinib.

### 3.3 Rank probabilities

**FIGURE 3:**
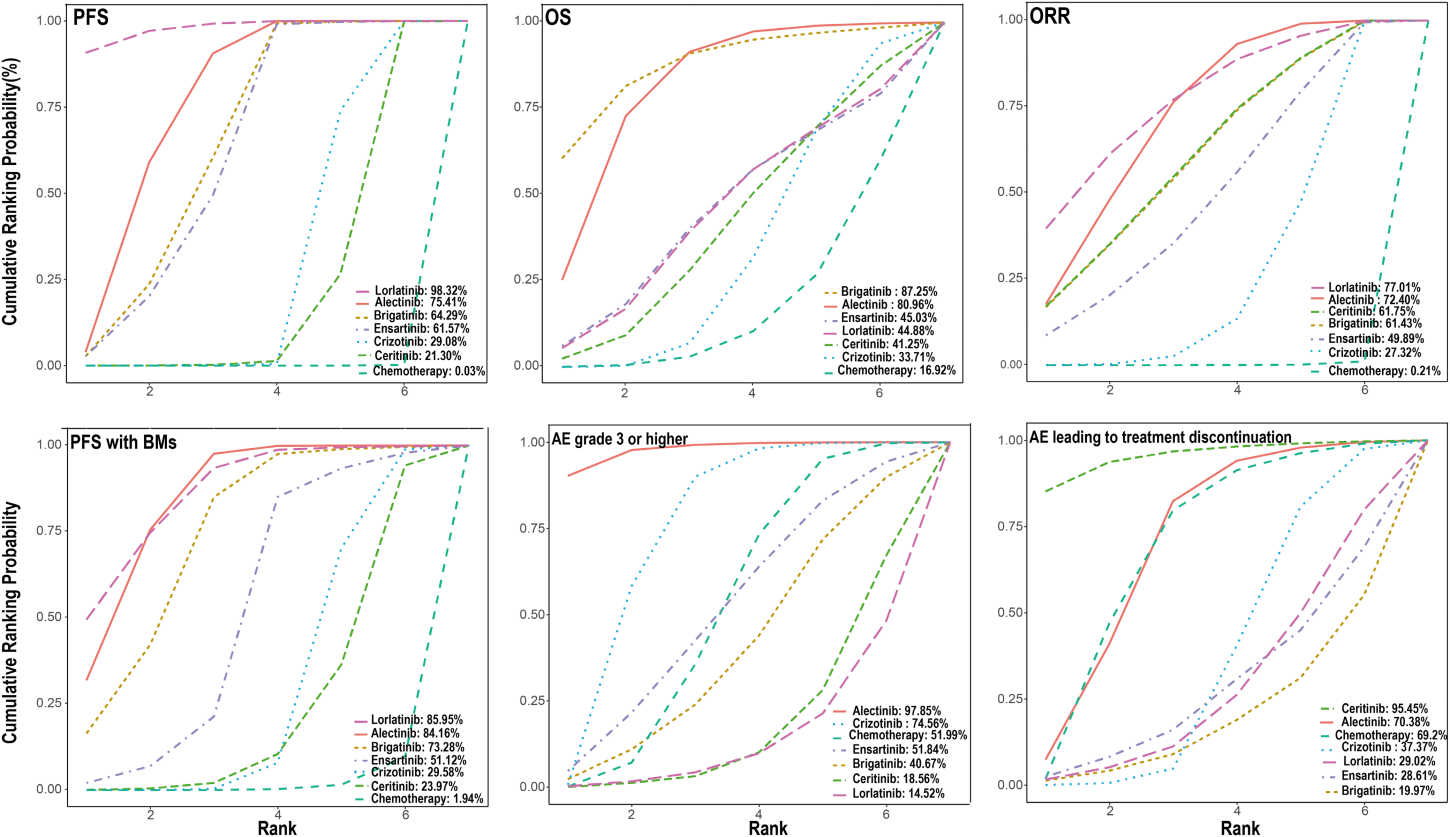
SUCRA was used to indicate the potential ranking probability of results on each treatment and identify the optimal treatment. Profiles show probability ranking for different kinds of treatments on PFS, OS, ORR, PFS with BM, and AEs (grade ≥ 3 and AEs leading to treatment discontinuation).

The results of the SUCRA are presented in Figure 3, which shows the potential ranking probability of various outcomes (detailed ranking results are summarized in Supporting Information Table S1). From the ranking of the primary outcome, lorlatinib was associated with outstanding PFS (cumulative probability: 98.93%), ORR (77.01%), and PFS with BM (85.95%). For patients with ALK‐positive NSCLC, brigatinib was better than the other drugs in terms of OS (87.25%). For SAEs, we showed that alectinib (97.85%) was significantly superior to the other ALKis. The probability for lorlatinib still ranked first in causing SAEs, followed by ceritinib. In the case of AEs leading to discontinuation, ceritinib (95.45%) and alectinib (70.38%) exhibited relatively high rankings. For safety with the lowest ranking, lorlatinib and brigatinib should be considerably noted. Moreover, it seemed that alectinib performed well with respect to both efficacy and safety.

### 3.4 Heterogeneity and inconsistency assessment

We observed acceptable heterogeneity in the comparison of all of the treatments by comparing the RE and FE models via a Bland–Altman plot, which showed that the estimated effects of the FE and RE models were similar for all of the outcomes (Supporting Information Figure S2). Supporting Information Figure S3 shows forest plots of pairwise comparisons with heterogeneity estimates. According to our assessment, half of all of the comparisons in the overall and subgroup populations had minimal (*I* ^2^ = 0%) or low heterogeneity. Additionally, high heterogeneity (*I* ^2^ > 50%) was observed in the following comparisons:

- Alectinib versus other treatments for PFS with BM (70.5%).
- Crizotinib versus chemotherapy for SAEs (79.5%).

In comparison with the inconsistency model, the fit of the consistency model performed similarly or better (Supporting Information Table S2). Consistency between direct and indirect evidence was observed after comparing results from pairwise and network meta‐analyses (Supporting Information Figure S4) to evaluate local inconsistencies. The node‐splitting analysis indicated that no significant heterogeneity or inconsistency occurred in the direct and indirect evidence. Summary, homogeneity, and consistency assumptions were confirmed.

### 3.5 Sensitivity analysis

**FIGURE 4:**
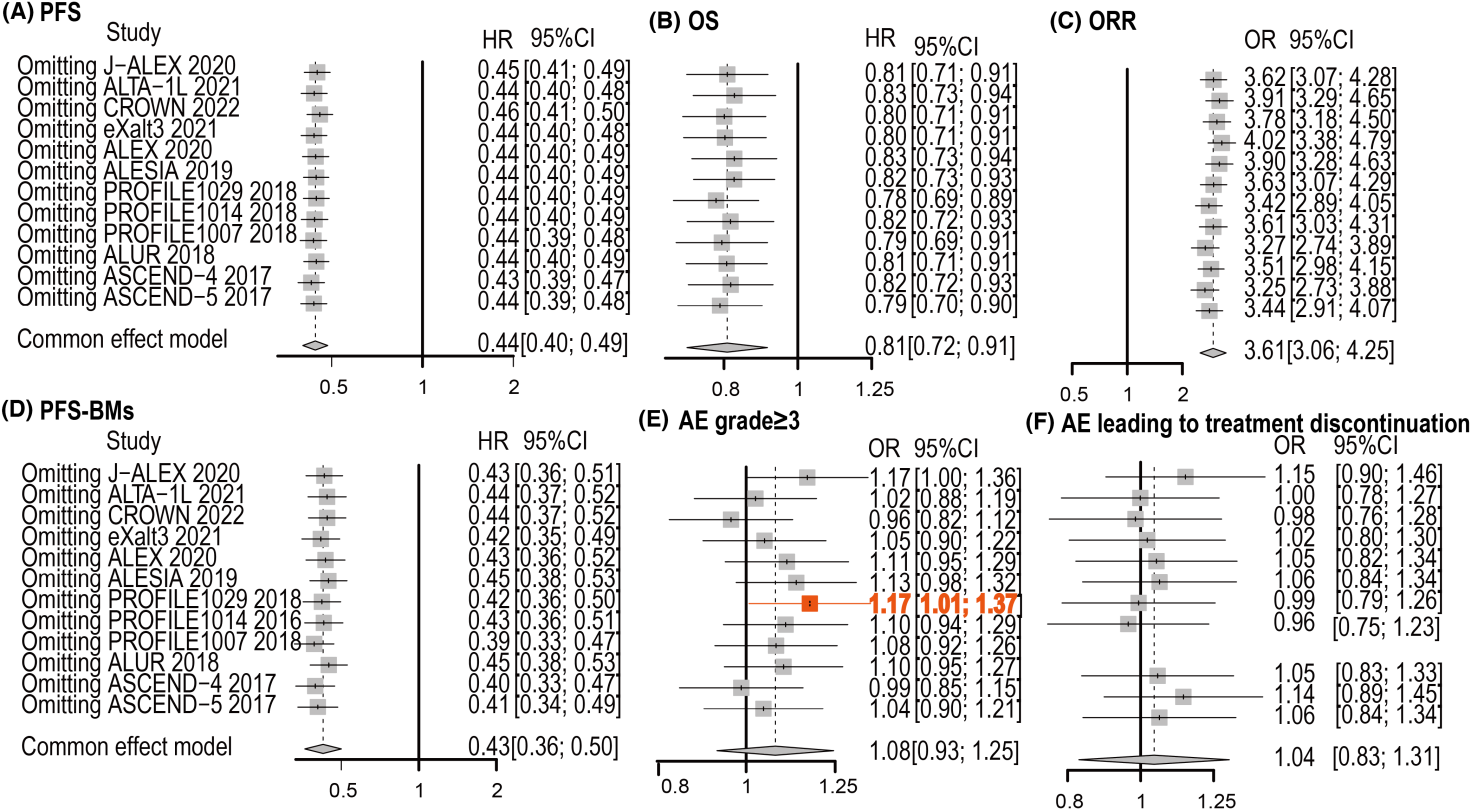
The sensitivity analysis was conducted by excluding each of the studies. (A) PFS; (B) OS; (C) ORR; (D) PFS‐BM; (E) AE grade ≥ 3; (F) AEs leading to treatment discontinuation.

**FIGURE 5:**
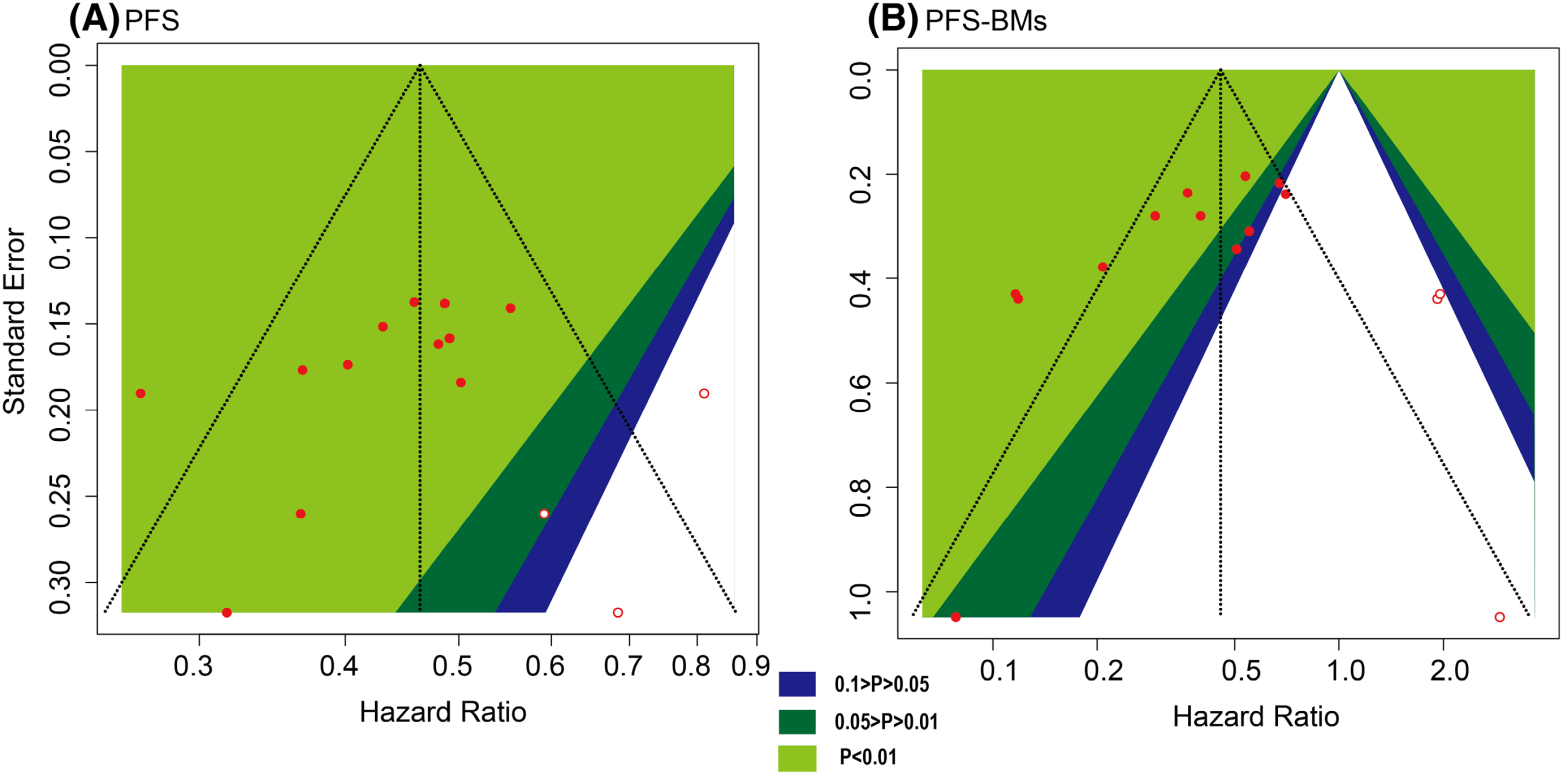
The contour‐enhanced funnel plot of the studies after using the trim and fill method. (A) PFS; (B) PFS‐BM.

We excluded each of the studies in the sensitivity analysis (Figure 4). The PROFILE1029 trial seemed to excessively influence the pooled estimate of grade 3 or higher AEs in the study removal analysis. After the exclusion of PROFILE1029 from the main analysis, AE grade 3 or higher was different. This finding could be observed in both the network meta‐analysis (OR: 1.17, 1.01 to 1.37) and standard pairwise meta‐analysis (OR: 1.08, 0.93 to 1.25; Figure 5). The PROFILE1029 trial may be a potential source of heterogeneity. The results after deleting the PROFILE1029 trial did not show relevant deviations compared with the conclusions of the original analysis (Supporting Information Table S3), which verified the robustness of the results. However, the significant differences in alectinib and crizotinib and alectinib and chemotherapy regarding OS disappeared. The same outcomes were found compared with crizotinib and brigatinib and lorlatinib and ceritinib regarding PFS with BM. The results revealed a higher probability of ranking the best PFS‐BM for lorlatinib. However, the significant toxicity between lorlatinib and crizotinib (OR: 2.15, 1.17 to 3.99) and between brigatinib and alectinib (OR: 2.28, 1.17 to 4.71) became more notable (Supporting Information Table S4).

### 3.6 Publication bias

Overall, there was no significant symmetry for any of the endpoints on visual estimations of comparison‐adjusted funnel plots. (Supporting Information Figure S6). Egger's test showed a potential publication bias among the PFS (*p* = 0.040) and PFS with BM (*p* = 0.006) datasets (Supporting Information Table S5). Hence, contour‐enhanced funnel plots and the trim‐and‐fill method were applied to estimate and adjust for the number and outcomes of the missing studies in PFS and PFS with BM (Figure 5). The results suggested that one of the three filled studies was in the region of nonstatistical significance, as indicated by squares, which illustrated that the main reason for the previously verified bias was high heterogeneity rather than publication bias (Figure 5A) in the PFS dataset. The filled risk estimate (HR: 0.46, 95% CI: 0.51 to 0.53) was still positive, as it was before (HR: 0.44, 95% CI: 0.39 to 0.49), indicating that the pooled HR was stable in our study. For the PFS‐with BM dataset, the contour‐enhanced funnel plot (Figure 5B) was also reasonably symmetrical (Egger's test: 0.6904) after imputing a total of 3 “missing” studies. Crucially, the “missing” area was contained within the area where nonsignificant studies would be located, which was inside of the triangle designated by *p* = 0.10 contour boundaries. This finding further supports the hypothesis that publication bias led to the observed asymmetry. The filled risk estimate (HR: 0.46, 95% CI: 0.29 to 0.70) was still positive, as it was before (HR: 0.36, 95% CI: 0.25 to 0.51), indicating that the pooled HR was also stable.

## 4 DISCUSSION

Our network meta‐analysis summarizes the latest information and provides a proposed ranking of PFS, OS, ORR, PFS with BM, SAEs, and AEs leading to treatment discontinuation for all of the marketed ALKis in ALK‐positive NSCLC. To ensure the high quality of the evidence, only phase III RCTs were adopted. A network analysis was applied to compensate for the lack of face‐to‐face RCTs on comparisons. Moreover, investigations on network heterogeneity and inconsistency were sufficiently explored. However, no face‐to‐face comparison RCTs have been conducted to confirm the superiority of all treatments in ALK‐positive NSCLC. Our study provides reliable and feasible suggestions to help professionals and ALK‐positive NSCLC patients make better decisions on their treatment.

To determine the most beneficial treatment for ALK‐positive NSCLC and evaluate the efficacy and safety of ALKis, several meta‐analyses have been performed. A recent meta‐analysis revealed that partial ALKis (alectinib, brigatinib, lorlatinib, and ensartinib) perform better with respect to PFS and ORR than crizotinib. With regard to SUCRA ranking analysis, lorlatinib ranked first, followed by alectinib, brigatinib, and ensartinib.^30^ Our meta‐analysis was different from the study by Cheng‐Hao Chuang and colleagues in several ways. First, our study aimed to evaluate all of the treatments using data from RCTs, whereas the study by Cheng‐Hao Chuang and colleagues did not include ceritinib and chemotherapy. Second, the previous study did not include information on OS, PFS with BM, and AEs leading to discontinuation, which prevented the extrapolation of treatment ranking to long‐term outcomes. In addition, the final results of ALTA‐1 L compared with brigatinib and crizotinib were updated in September 2021. The two large RCTs (eXalt3 and CROWN) that compared ensartinib or lorlatinib with crizotinib were also separately updated in 2022 and 2021. Third, our study was conducted by using the Bayesian framework, whereas the recent study used the frequentist framework. In contrast to frequentist analysis, Bayesian analysis is more in line with our natural pattern of inference, which incorporates prior information and is able to compute probabilities for different hypotheses directly from the posterior distribution.^31^ Moreover, in the case of high heterogeneity and sparse data, that is, one or two trials per contrast, the Bayesian method has higher coverage and accuracy than the frequentist analysis.^32^ Finally, we used a random effects model as well because it could be the most appropriate and conservative methodology to account for between‐trial heterogeneity within each comparison, whereas the study by Cheng‐Hao Chuang and colleagues used the fixed effect model. The assumption of a fixed effect model had no (or a negligible amount of) heterogeneity and the same true effect size for all of the trials, which was recognized to be typically unrealistic.^33^ Hence, a Bayesian network meta‐analysis with a conservative random effects model is urgently required to rank all treatments in ALK‐positive NSCLC with regard to effectiveness and safety concerns.

All ALKis have been suggested to be comparatively better than chemotherapy for ALK‐positive NSCLC in PFS and ORR, which was predominantly consistent with conclusions from previous studies. Lorlatinib demonstrated the longest PFS and highest ORR with the highest SUCRA ranking, followed by alectinib. With regard to ORR, alectinib ranked second, with brigatinib ranking second in a previous article.^30^ The OS improvement was also controversial. We did not observe any significant improvements in OS among the include studies, while OS with alectinib over crizotinib or chemotherapy was significantly improved. Brigatinib demonstrated the longest OS with the highest SUCRA ranking, followed by alectinib, but there was no significant difference between ALKis. The findings may have been affected by the crossover between treatments following disease progression. In addition, there was a considerable difference in the duration of RCT treatment. It seemed that participants exposed to ALKis had enormously longer durations than those exposed to chemotherapy, likely caused by crossover to an ALKi after disease progression. Crossover between the treatment groups was permitted in five of the 12 Trials (ALTA‐1,^23^ PROFILE 1029,^26^ ALUR,^27^ ASCEND‐4,^28^ ASCEND‐5^29^). Such crossovers might result in the OS of patients receiving an ALKi being greater than that of patients receiving chemotherapy or crizotinib alone. This unconformity highlighted that the treatment exposure duration was different between patients randomized to chemotherapy and ALKis, which allowed for crossover. For instance, in the ALUR trial, the median treatment exposure duration was approximately 20.1 weeks for alectinib in comparison to 6 weeks for chemotherapy. The same condition was found in the ASCEND‐4 trial, in which the median treatment exposure duration was approximately 66.4 weeks for ceritinib and 29.9 weeks for chemotherapy. This outcome was consistent with clinical experience. It was proposed that ALKis had a favourable effect on patients who had previous treatment experiences. Indeed, longer PFS and improved quality of life have been found with alectinib or crizotinib (ALUR) and with crizotinib after disease progression on chemotherapy (PROFILE 1014^4^), even though OS showed no statistically significant differences. The CROWN^14^ and eXalt3^15^ trials were ongoing, which affects the efficacy comparison in terms of OS. Therefore, the conclusion that alectinib possessed an advantage over lorlatinib and ensartinib in overall survival needs to be revalidated when additional data are available with longer follow‐ups.

Unlike chemotherapy and crizotinib, all of the other ALKis are CNS penetrants. In ALK‐positive NSCLC with CNS metastases, Binghao Zhao et al.^34^ believed that for BM patients in PFS, lorlatinib showed the strongest benefit, but ORR differences were not obvious between third‐ and second‐generation inhibitors. However, the novel third‐generation ALKi ensartinib with CNS activity^35^ and PFS with BM were not included in this study. In our subgroup analysis of the baseline brain metastasis patients, lorlatinib had the strongest statistical superiority in terms of PFS compared with other ALKis, followed by alectinib. This result was supported by the study by Binghao Zhao and colleagues.^34^ This study also indicated that brigatinib was related to an even better ORR for BM patients, followed by lorlatinib. Unfortunately, ORR was not included in our study. The strength of lorlatinib arose from its novel mechanism. Lorlatinib has high membrane and blood–brain barrier permeability and can pass through the blood–brain barrier.^36^ In addition, alectinib had a similar mechanism as lorlatinib. For PFS for BM patients, no previous studies have made comparisons with all ALKis. In the eXalt3 trial, ensartinib had a longer PFS in the baseline BM population than crizotinib, which was also supported by our study. However, ensartinib was not superior to alectinib, brigatinib, or lorlatinib in terms of PFS with BM. Compared with ceritinib, we observed improved PFS with alectinib and brigatinib, which showed no significant difference between brigatinib and ceritinib.^37^ In terms of PFS with BM, brigatinib showed no advantage over alectinib and lorlatinib in our study. Few studies have evaluated the efficacy of ALKis in NSCLC without CNS metastases, which precludes a network meta‐analysis. Only three of 12 trials (ALTA‐1 L, eXalt3, CROWN) have stratified statistics for patients without BM. In the ALTA‐1 L study, brigatinib significantly extended overall PFS compared to crizotinib (HR: 0.65, 0.44 to 0.97) in patients without baseline brain metastases. Compared with crizotinib, ensartinib reduced the risk of central progression in patients without baseline BM (HR 0.32, 0.16 to 0.63) in the eXalt3 study. In contrast to crizotinib, the intracranial efficacy of lorlatinib was particularly prominent in the CROWN study. The 3‐year intracranial progression‐free rate in patients without baseline BM was 99.1% in the first‐line lorlatinib group, achieving virtually no brain metastasis. Thus, it could be roughly concluded that second‐ and third‐generation inhibitors were effective not only in NSCLC patients with BM but also in patients without BM compared to first‐generation inhibitors.

Adverse effects are significantly important to ALKi selection. However, the SAEs of ALKis cannot be ignored. With regard to antitumor efficacy and safety, alectinib had superior performance compared with crizotinib.^38^ The incidence of SAEs was more than 25% in ALKi‐treated patients in the study by Hou H and colleagues (crizotinib at 38.09%, alectinib at 26.24%, ceritinib at 41.44%, and brigatinib at 41.68%).^39^ In our study, although lorlatinib demonstrated excellent effectiveness, it was related to the highest SAE rate. Hence, alectinib demonstrated the safest outcome, which was consistent with the results of a previous study.^39^ However, first‐ and second‐generation ALKis were consistent with chemotherapy in the risk of SAEs.^40^ Interestingly, chemotherapy was safer than ensartinib, brigatinib, ceritinib, and lorlatinib, although no significant difference was found in our study.

Each ALKi had unique SAEs in all of the patients. Qinghua Zeng et al.^38^ reported that more nervous system disorders, gastrointestinal disorders, and eye disorders were found with crizotinib than with alectinib. Moreover, alectinib was accompanied by liver enzyme elevation, and these disorders covered increasing levels of ALT (3.7%), AST (3.7%), and bilirubin (3.2%).^41^ However, Wei Tian et al.^42^ reported that the frequencies of grade ≥ 3 hepatotoxicity induced by ceritinib were higher than those induced by alectinib or crizotinib in advanced NSCLC. The use of ceritinib is still limited due to serious gastrointestinal AEs. Specifically, the risk of diarrhoea increased to 61% among patients, with grade ≥ 3 diarrhoea being observed in 2% of all trials. In addition, brigatinib was observed to result in early‐onset pulmonary events and led to a significantly higher incidence rate of pneumonitis (OR: 4.827, p = 0.007) than alectinib, as reported by Hyun Suha et al.^43^ However, this finding should be interpreted with caution, because only one phase 2 study was available. The most common adverse event of ensartinib was rash (56%), and 23% of patients had grade 3 AEs, which was not common in other ALKis. The distribution of ALKis is mainly in the epidermis. Nevertheless, the concentrations of ensartinib in the skin were nine times higher than those in the plasma, which could cause a high frequency of rash.^44^ The rashes were manageable and consisted mostly of grade 1 or 2 events. In addition, no grade 4 AEs were reported, and no patients were discontinued due to rash. Most SAEs in lorlatinib were not serious and life‐threatening metabolic AEs (hypercholesterolemia at 16%, hypertriglyceridemia at 20%, and increased weight at 17%).^7^

In the PROFILE 1029 trial, the permanent discontinuation of crizotinib was associated with AEs in 3 of 19 patients (anemia, death, and interstitial lung disease). In the chemotherapy arm, there was one of 4 permanent discontinuations (4.0%). Furthermore, a minority of patients discontinued due to AEs suspected to be related to the study drug in both groups (15% of ceritinib group vs. 11% of chemotherapy).^28^ Furthermore, 6 (10%) crizotinib‐treated patients and 9 (7%) alectinib‐treated patients discontinued due to AEs, including interstitial lung disease, liver injury, and abnormal hepatic function.^25^ Although the alectinib treatment duration was longer than that of chemotherapy (20.1 weeks versus 6.0 weeks), the probability of AEs leading to discontinuation was lower with alectinib (5.7%) than with chemotherapy (8.8%).^27^ Treatment was discontinued due to the presence of AEs in 13% versus 9% of treated patients in brigatinib and crizotinib, respectively.^23^ Additionally, lorlatinib was associated with a high incidence of SAEs. However, the permanent treatment discontinuation rate was similar (7% in lorlatinib vs. 9% in crizotinib).^14^ Thirteen (9.09%) ensartinib‐treated patients and 10 (6.85%) crizotinib‐treated patients discontinued treatment because of AEs, including increased liver enzymes and pneumonitis.^15^ However, there has been no systematic attempt to synthesize these data, and the difference in AEs leading to discontinuation by ALKis has yet to be clarified. In our study, brigatinib was found to be the worst in terms of tolerability owing to AEs leading to discontinuation.

The overall results remained relatively robust, which can be concluded from the sensitivity analysis. The loss of superiority over alectinib versus crizotinib and alectinib versus chemotherapy regarding OS only included PROFILE1029 trials. The same results were found in PFS with BM compared to crizotinib versus brigatinib and lorlatinib versus ceritinib. However, the significant toxicity between lorlatinib and alectinib or brigatinib and crizotinib became more notable.

There were several limitations of our study. First, NSCLC patients with ALK mutations tend to have long survival times. The current meta‐analysis only included certain clinical trials, which might lead to immature OS results, which impacted the efficacy comparison. For instance, the CROWN and eXalt3 trials are still being conducted. Bias in the evaluation of the drug might occur due to the evolving OS data. Therefore, the conclusion that we obtained in this study is that alectinib is superior to lorlatinib in terms of OS. The outcome of ensartinib needs to be updated when longer follow‐ups could bring further survival data access to the research. Second, the results were explained by the limited total sample size. The number of large clinical trials, especially clinical trials designed for patients with BM, is expected to increase. The conclusion of PFS with BM in this study could only be assessed by subgroup analysis with insufficient information. OS, ORR, and AEs of BM patients, all these data were limited. Third, potential publication and selection biases still existed in the study. Small study effects (small number of trials that were included in each comparison) and publication bias were observed in the PFS with BM dataset. All of the patients who accepted the interventions were included in this research, whether they received former therapy or not. A potential survival bias could occur because the results were collected from data instead of first‐line therapy observations, and resistance might exist in some patients.

## 5 CONCLUSION

ALK‐positive NSCLC patients treated with ALKis are likely to have longer PFS and a higher ORR than those treated with chemotherapy. PFS with or without BM may be improved by lorlatinib and alectinib. Moreover, the benefit of lorlatinib in terms of OS did not exceed that of alectinib, and lorlatinib had the highest risk of SAEs. However, it is necessary to interpret these results with caution, and the assessment of OS is likely confounded by treatment crossover. Furthermore, alectinib was indicated as a preferable ALK in ALK‐positive NSCLC. The latest ALKi, known as ensartinib, did not provide any benefits in terms of efficacy or safety. Future face‐to‐face trials to assess the relative efficacy of all ALKis are warranted, and more studies are needed to verify these conclusions.

## AUTHOR CONTRIBUTIONS

**Bei Zheng:** Formal analysis (lead); software (lead); writing – original draft (lead); writing – review and editing (equal). **Hong Jiang:** Formal analysis (supporting); writing – original draft (supporting); writing – review and editing (equal). **Wenjuan Yang:** Data curation (equal); formal analysis (supporting). **Ying Li:** Data curation (equal); software (supporting). **Bingqing Liang:** Writing – original draft (supporting); writing – review and editing (supporting). **Jun Zhu:** Validation (equal). **Nanmei Chen:** Validation (equal). **Miao Chen:** Methodology (supporting). **Meiling Zhang:** Conceptualization (lead); methodology (lead); project administration (lead); supervision (lead).

## FUNDING INFORMATION

The study was supported by the Basic Public Welfare Research Program of Zhejiang Province (LGF20G030004).The Natural Science Foundation of Zhejiang Provincial (LYQ20H310003).

## CONFLICT OF INTEREST STATEMENT

No authors declared a conflict of interest.

## Supporting information


Data S1.

## Data Availability

All data generated or analyzed during this study are included in this published article and its supplementary information files.
