# Supplementary material for: A Bayesian network meta‐analysis of ALK inhibitor treatments in patients with ALK‐positive non‐small cell lung cancer
Source: Cancer Med. 2023 Jun 19;12(15):15983–97. doi: 10.1002/cam4.6241 (PMC10469807; doi:10.1002/cam4.6241)
Supplement: Supplementary file 1 — Data S1. [file CAM4-12-15983-s001.doc]

**A** **Bayesian network meta analysis of ALK inhibitor treatments in patients with** **ALK-positive non-small-cell lung cancer(NSCLC)**

**Supplementary materials**

[Appendix 1: Search strategy 2](#__RefHeading___Toc18202)

[Appendix 2: Risk of bias assessment 4](#__RefHeading___Toc23823)

[Figure S1: Convergence of the three chains established by inspection of the history feature and the Brooks-Gelman-Rubin diagnostic 17](#__RefHeading___Toc24677)

[Figure S2: Bland Altman plot 20](#__RefHeading___Toc12909)

[Figure S3: Forest plots depicting results of head-to-head comparisons according to pairwise meta-analyses 21](#__RefHeading___Toc25894)

[Figure S4: Forest plots depicting results of head-to-head comparisons in according to pairwise and network meta-analyses. 22](#__RefHeading___Toc23483)

[Figure S5:Node-splitting analysis of inconsistency. 22](#__RefHeading___Toc28721)

[Figure S6: Comparison-adjusted funnel plots for the primary endpoints and adverse events. 23](#__RefHeading___Toc7391)

[Table S1. Bayesian ranking results and SUCRA of network meta-analysis. 24](#__RefHeading___Toc8338)

[Table S2: Comparisons of the fit of consistency and inconsistency models using deviance information criteria (DIC). 26](#__RefHeading___Toc24692)

[Table S3: Pooled estimates of the second sensitivity analysis stratifying patients by excluded the PROFILE 1029 study. 26](#__RefHeading___Toc18252)

[Table S4: SUCRA results of the second sensitivity analysis stratifying patients by excluded the PROFILE 1029 study. 27](#__RefHeading___Toc18681)

[Table S5: The Egger’s and Begg’s test of the primary endpoints and adverse events. 27](#__RefHeading___Toc25670)

[References 28](#__RefHeading___Toc10387)

# Appendix 1: Search strategy

**Pubmed**

((((((((((((((((((((((((((((EML4-ALK) OR EML4-ALK fusion gene) OR EML4-ALK fusion-positive) OR EML4-ALK positive) OR EML4-ALK-positive) OR EML4-ALK translocation) OR EML4-ALK rearrangement) OR anaplastic lymphoma kinase (ALK) gene arrangements) OR anaplastic lymphoma kinase-positive) OR anaplastic lymphoma kinase positive) OR anaplastic lymphoma kinase fusions) OR anaplastic lymphoma kinase-fusions) OR anaplastic lymphoma kinase-translocated) OR anaplastic lymphoma kinase translocated) OR ALK translocation) OR ALK-positive) OR ALK positive) OR ALK-rearranged) OR ALK rearranged) OR ALK rearrangements) OR ALK-rearrangements) OR ALK fusion) OR ALK compound mutation) OR ALK)) AND ((((((((("Carcinoma, Non-Small-Cell Lung"[Mesh]) OR ((Carcinoma) AND Non Small Cell Lung)) OR ((Carcinomas) AND Non-Small-Cell Lung)) OR ((Lung Carcinoma) AND Non-Small-Cell)) OR ((Lung Carcinomas) AND Non-Small-Cell)) OR ((Carcinoma) AND Non-Small Cell Lung)) OR (((((Non-Small Cell Lung Cancer) OR Non-Small-Cell Lung Carcinomas) OR Nonsmall Cell Lung Cancer) OR Non-Small-Cell Lung Carcinoma) OR Non Small Cell Lung Carcinoma)) OR NSCLC)))) AND (Search (((((((Anaplastic lymphoma kinase inhibitors) OR Anaplastic lymphoma kinase inhibitor) OR ALK inhibitor) OR ALK inhibitors) OR ALK-inhibitor) OR ALK-inhibitors)) OR ((((((("lorlatinib" [Supplementary Concept]) OR (“Ensartinib”[Supplementary Concept] OR X-396) OR (((("7-amino-12-fluoro-2,10,16-trimethyl-15-oxo-10,15,16,17-tetrahydro-2H-8,4-(metheno)pyrazolo(4,3-h)(2,5,11)benzoxadiazacyclotetradecine-3-carbonitrile") OR PF-06463922) OR PF06463922) OR Lorbrena))) OR (("ceritinib" [Supplementary Concept]) OR ((((“5-chloro-N2-(2-isopropoxy-5-methyl-4-(piperidin-4-yl)phenyl)-N4-(2-(isopropylsulfonyl)phenyl)pyrimidine-2,4-diamine”) OR zykadia) OR LDK378)))) OR (("brigatinib" [Supplementary Concept]) OR ((Alunbrig) OR AP26113))) OR (("alectinib" [Supplementary Concept]) OR (((Alecensa) OR RO5424802) OR CH5424802))) OR (("Crizotinib"[Mesh]) OR (((((((xalkori) OR PF02341066) OR PF-02341066) OR PF-2341066) OR PF 2341066) OR PF2341066) OR PF 02341066))))) AND ((((((((((((((((((Meta-Analysis as Topic/) OR ("meta"[All Fields] AND " analy$"[All Fields])) OR meta analy*[All Fields])) OR metaanaly*[All Fields])) OR (systematic[Title/Abstract] AND (review*[All Fields] or overview*[All Fields])))) OR "Review" [Publication Type])) OR (("review literature as topic"[MeSH Terms] OR ("review"[All Fields] AND "literature"[All Fields]) OR "review literature as topic"[All Fields])))) OR Review/)) NOT (((animal/)) NOT ((animal/) AND human/))))) OR ((((("Randomized Controlled Trials as Topic"[Mesh]) OR ((Clinical Trials) AND Randomized)) OR ((Trials) AND Randomized Clinical)) OR ((Controlled Clinical Trials) AND Randomized)) OR ((Randomized) AND Controlled Trials) Filters: Humans))

**Embase.com**

((carcinoma:ab,ti AND 'non small cell lung':ab,ti) OR ('lung carcinoma':ab,ti AND 'non small cell':ab,ti) OR (carcinoma:ab,ti AND 'non small cell lung':ab,ti) OR ('non-small-cell lung carcinoma':ab,ti OR 'non small cell lung carcinoma':ab,ti OR 'non-small-cell lung carcinomas':ab,ti OR 'non-small cell lung cancer':ab,ti OR nsclc:ab,ti) OR (carcinomas:ab,ti AND 'non-small-cell lung':ab,ti)) **AND** ('eml4 alk':ab,ti OR alk:ab,ti OR 'alk-':ab,ti OR 'anaplastic lymphoma kinase':ab,ti OR 'anaplastic lymphoma kinase-':ab,ti OR 'anaplastic lymphoma kinase (alk)':ab,ti) AND (('anaplastic lymphoma kinase inhibitors':ab,ti OR 'anaplastic lymphoma kinase inhibitor':ab,ti OR

'alk inhibitor':ab,ti OR 'alk inhibitors':ab,ti OR 'alk-inhibitor':ab,ti OR 'alk-inhibitors':ab,ti) OR('crizotinib':ab,ti OR 'xalkori':ab,ti OR 'pf02341066' OR 'pf-02341066' OR 'pf-2341066' OR

'pf 2341066' OR 'pf2341066' OR 'pf 02341066') OR ('alectinib':ab,ti OR 'alecensa':ab,ti OR 'ro5424802' OR 'ch5424802') OR ('brigatinib':ab,ti OR 'alunbrig':ab,ti OR 'ap26113') OR ('ceritinib':ab,ti OR '5-chloro-n2-(2-isopropoxy-5-methyl-4-(piperidin-4-yl)phenyl)-n4-(2-(isopropylsulfonyl)phenyl)pyrimidine-2,4-diamine' OR 'zykadia':ab,ti OR 'ldk378') OR ('lorlatinib':ab,ti OR '7-amino-12-fluoro-2,10,16-trimethyl-15-oxo-10,15,16,17-tetrahydro-2h-8,4-(metheno)pyrazolo(4,3-h)(2,5,11)benzoxadiazacyclotetradecine-3-carbonitrile' OR 'pf-06463922' OR 'pf06463922' OR

'lorbrena':ab,ti)OR (‘Ensartinib’:ab,ti) OR (‘X-396’:ab,ti)) **AND** ('clinical trials':ab,ti AND randomized:ab,ti OR (trials:ab,ti AND 'randomized clinical':ab,ti) OR 'randomized controlled trials':ab,ti OR ('controlled clinical trials':ab,ti AND randomized:ab,ti) OR (randomized:ab,ti AND 'controlled trials':ab,ti) OR rcts:ab,ti)

**Medline**

((((((MHX=(Carcinoma, Non-Small-Cell Lung)) OR TI=(Non-Small Cell Lung Cancer)) OR TI=(Non$Small-Cell Lung Carcinoma)) OR TI=(Carcinoma, Non-Small Cell Lung)) AND TI=((((((((((((((((((((((((EML4-ALK) OR EML4-ALK fusion gene) OR EML4-ALK fusion-positive) OR EML4-ALK positive) OR EML4-ALK-positive) OR EML4-ALK translocation) OR EML4-ALK rearrangement) OR anaplastic lymphoma kinase (ALK) gene arrangements) OR anaplastic lymphoma kinase-positive) OR anaplastic lymphoma kinase positive) OR anaplastic lymphoma kinase fusions) OR anaplastic lymphoma kinase-fusions) OR anaplastic lymphoma kinase-translocated) OR anaplastic lymphoma kinase translocated) OR ALK translocation) OR ALK-positive) OR ALK positive) OR ALK-rearranged) OR ALK rearranged) OR ALK rearrangements) OR ALK-rearrangements) OR ALK fusion) OR ALK compound mutation) OR ALK)) AND AB=((((((((Anaplastic lymphoma kinase inhibitors) OR Anaplastic lymphoma kinase inhibitor) OR ALK inhibitor) OR ALK inhibitors) OR ALK-inhibitor) OR ALK-inhibitors)) OR ((((((("lorlatinib" [Supplementary Concept]) OR (((("7-amino-12-fluoro-2,10,16-trimethyl-15-oxo-10,15,16,17-tetrahydro-2H-8,4-(metheno)pyrazolo(4,3-h)(2,5,11)benzoxadiazacyclotetradecine-3-carbonitrile") OR PF-06463922) OR PF06463922) OR Lorbrena))) OR (("ceritinib" [Supplementary Concept]) OR ((((“5-chloro-N2-(2-isopropoxy-5-methyl-4-(piperidin-4-yl)phenyl)-N4-(2-(isopropylsulfonyl)phenyl)pyrimidine-2,4-diamine”) OR zykadia) OR LDK378)))) OR (("brigatinib" [Supplementary Concept]) OR ((Alunbrig) OR AP26113))) OR (("alectinib" [Supplementary Concept]) OR (((Alecensa) OR RO5424802) OR CH5424802))) OR (("Crizotinib"[Mesh]) OR (((((((xalkori) OR PF02341066) OR PF-02341066) OR PF-2341066) OR PF 2341066) OR PF2341066) OR PF 02341066))))) AND AB=((((("Randomized Controlled Trials as Topic"[Mesh]) OR ((Clinical Trials) AND Randomized)) OR ((Trials) AND Randomized Clinical)) OR ((Controlled Clinical Trials) AND Randomized)) OR ((Randomized) AND Controlled Trials))

**Cochrane CENTRAL**

((MeSH descriptor: [Carcinoma, Non-Small-Cell Lung] explode all trees) OR ((Carcinoma):ti,ab,kw AND (Non-Small Cell Lung):ti,ab,kw ) OR ((Lung Carcinoma):ti,ab,kw AND (Non-Small-Cell):ti,ab,kw) OR ((Lung Carcinomas):ti,ab,kw AND (Non-Small-Cell):ti,ab,kw) OR ((Carcinoma):ti,ab,kw AND (Non Small Cell Lung):ti,ab,kw)) **AND** ((("ALK-positive "):ti,ab,kw OR (ALK):ti,ab,kw OR ("ALK-rearranged"):ti,ab,kw)) OR (("anaplastic lymphoma kinase-*") OR ("anaplastic lymphoma kinase")) OR ((ALK):ti,ab,kw)) **AND** ((“Anaplastic lymphoma kinase inhibitor”) OR ("ALK inhibitor") OR (ALK-inhibitor) (MeSH descriptor: [Crizotinib] explode all trees) OR ((xalkori) OR (PF 2341066) OR (PF-2341066) OR ("PF-2341066") OR ("PF 02341066")) OR ((alectinib) OR (alecensa) OR (RO5424802) OR (CH5424802)) OR ((brigatinib) OR (alunbrig) OR (AP26113)) OR ((ceritinib) OR ( zykadia) OR (LDK378))OR (Ensartinib) OR (X-396))

**Clinicaltrials.gov**

(Non-Small Cell Lung) OR (Anaplastic lymphoma kinase inhibitor) OR (ALK inhibitor) OR (xalkori) OR (PF-2341066) OR (alectinib) OR (alecensa) OR (RO5424802) OR (CH5424802) OR ((brigatinib) OR (alunbrig) OR (AP26113) OR ((ceritinib) OR ( zykadia) OR (LDK378) OR Lorlatinib OR lorbrena OR PF-06463922 OR Brigatinib OR Alunbrig OR AP26113 OR Ensartinib OR X-396

# Appendix 2: Risk of bias assessment

Risk of bias was assessed by use of Cochrane’s risk of bias tool for RCTs that reported at least one outcome of interest

1. **Summary of risk of bias across studies**

**
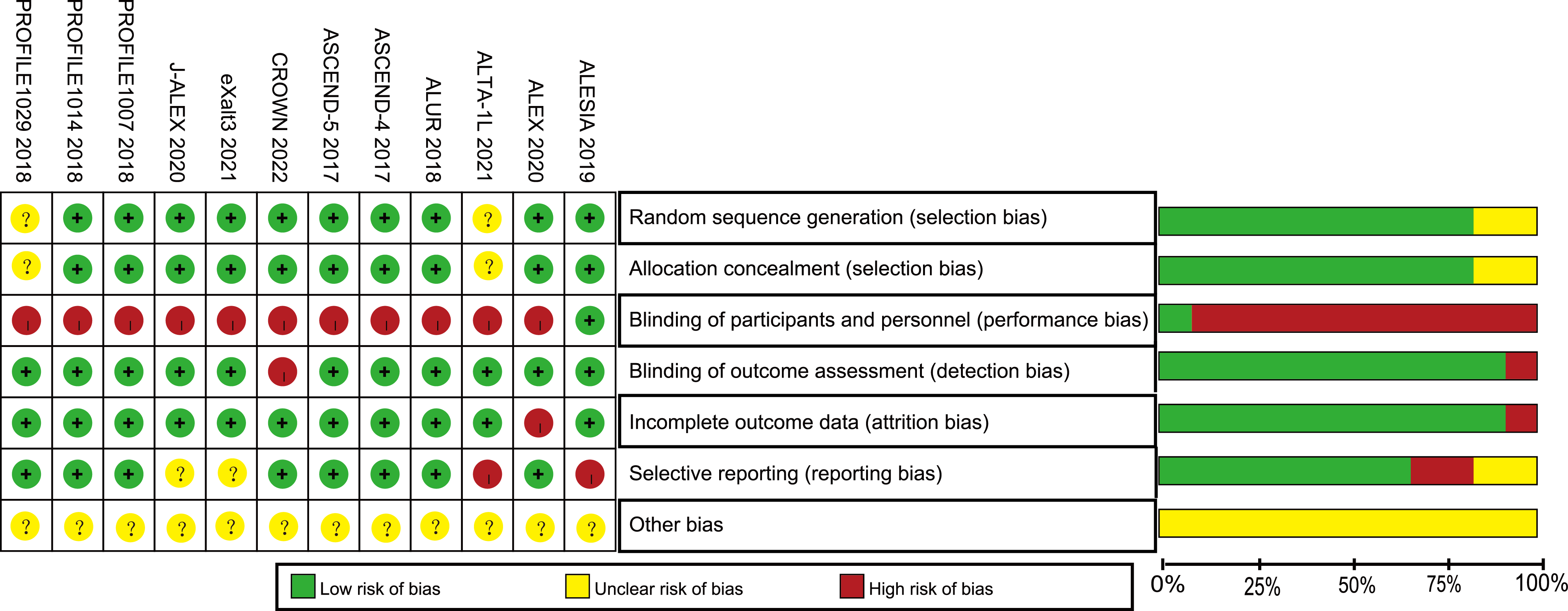
**

Summary of results from assessment of studies using the Cochrane risk of bias tool.

**B. Risk of bias assessment by domain**

| **Author, year** | **study** | **Sequence generation** | **Allocation concealment** | **Blinding, personnel/ participants** | Blinding of outcome assessment | **Incomplete outcome data** | **Selective reporting** | **Other threats** |
| --- | --- | --- | --- | --- | --- | --- | --- | --- |
| Nishio M 2018^[1]^ | PROFILE 1007 | Low | Low | High | Low | Low | Low | Unclear |
| Solomon B.J2018^[2]^ | PROFILE 1014 | Low | Low | High | Low | Low | Low | Unclear |
| Wu 2018^[3]^ | PROFILE 1029 | Unclear | Unclear | High | Low | Low | Low | Unclear |
| Alice T. Shaw2022^[4]^ | CROWN | Low | Low | High | High | Low | Low | Unclear |
| Leora Horn 2021^[5]^ | eXalt3 | Low | Low | High | Low | Low | Unclear | Unclear |
| Kazuhiko Nakagawa 2020^[6]^ | J-ALEX | Low | Low | High | Low | Low | Unclear | Unclear |
| T. Mok 2020[^[7]^ | ALEX | Low | Low | High | Low | High | Low | Unclear |
| Shaw 2017^[8]^ | ASCEND-5 | Low | Low | High | Low | Low | Low | Unclear |
| Soria 2017^[9]^ | ASCEND-4 | Low | Low | High | Low | Low | Low | Unclear |
| D.Ross Camidge2021^[10]^ | ALTA-1L | Unclear | Unclear | High | Low | Low | High | Unclear |
| Novello2018^[11]^ | ALUR | Low | Low | High | Low | Low | Low | Unclear |
| Zhou 2019^[12]^ | ALESIA | Low | Low | Low | Low | Low | High | Unclear |

**C. Detailed risk of bias assessment**

| Nishio M 2018^[1]^ **(PROFILE 1007;** **NCT00932893)** | | | |
| --- | --- | --- | --- |
| **Methods:** Open-label, multisite, randomized, phase 3 study | | | |
| **Population:  **18 yr, ALK-positive NSCLC, with ECOG score of 0–2, with progressive disease after one prior platinum-based chemotherapy regimen | | | |
| **Interventions:** Crizotinib (250 mg twice daily) in 3-wk cycles v. intravenous chemotherapy (pemetrexed 500 mg/m^2^ or docetaxel 75 mg/m^2^) | | | |
| **Cross-over between treatment groups:** Not during study period; participants from the chemotherapy arm could enroll in NCT00932451(PROFILE 1005). | | | |
| **Reported outcomes of interest to this review:**  Progression-free survival, as assessed by independent radiologic review.  Overall survival,  ORR  PFS with brain metastases  SAEs (an adverse event that results in death, is life threatening, requires inpatient hospitalization or extends a current hospital stay, results in ongoing or significant incapacity or interferes substantially with normal life functions, or causes a congenital anomaly or birth defect) | | | |
| **Risk of bias** | | | |
| **Domain** | **Judgment** | | **Support for judgment** |
| Random sequence generation | Low risk | | Centralized Interactive Voice Response System (IVRS)/website |
| Allocation concealment | Low risk | | Centralized allocation |
| Blinding of participants and personnel | High risk | | Open label study in which knowledge of group assignment might have influenced performance |
| Blinding of outcome assessment | Low risk | | Despite being open label, knowledge of group assignment would not be expected to affect objective outcomes assessment. A central blinded independent radiologic review was conducted for PFS and tumor Response  rates |
| Incomplete outcome data | Low risk | | Lost to follow-up: chemotherapy 2/174 (1%), crizotinib 4/173 (2%); withdrawn consent: chemotherapy 3/174 (2%), crizotinib 2/173 (1%); other reasons or protocol violations: chemotherapy 12/173 (7%), crizotinib 2/173 (1%). Unbalanced in terms of withdrawals due to “other reasons/protocol violations” in the chemotherapy group. However, given the comparatively low numbers and percentage of the whole population, the risk of attrition bias is judged to be low |
| Selective reporting | Low risk | | No detected difference in reporting between protocol and publication |
| Other potential threats to validity | Unclear | | Pharmaceutical funding (Pfizer)  Participants in the chemotherapy group with disease progression were permitted to receive crizotinib as part of an additional study (NCT00932451); however, the authors note that their analysis of overall survival was “likely confounded by the high crossover rate among patients in the chemotherapy group." |
| Solomon B.J2018**^[2]^ (PROFILE 1014; NCT01154140)** | | | |
| **Methods:** Open-label, multi-center, randomized, phase 3 study | | | |
| **Population:** Patients with advanced, non-squamous ALK-positive NSCLC | | | |
| **Interventions:** oral Crizotinib (250 mg twice daily) in 21 day cycles v. intravenous pemetrexed (500 mg/m^2^) or cisplatin (75 mg/m^2^) for up to six 21-day cycles | | | |
| **Cross-over between treatment groups:** Yes; participants in the chemotherapy arm with disease progression could cross to the crizotinib arm provided safety criteria were met | | | |
| **Reported outcomes of interest to this review:**  Progression-free survival (the time from randomization to RECISTdefined progression, as assessed by independent radiologic review, or death).  Objective response rate  Overall survival  PFS with brain metastases  SAEs (an adverse event that results in death, is life threatening, requires inpatient hospitalization or extends a current hospital stay, results in ongoing or significant incapacity or interferes substantially with normal life functions, or causes a congenital anomaly or birth defect)  AEs leading to discontinuation | | | |
| **Risk of bias** | | | |
| **Domain** | **Judgment** | | **Support for judgment** |
| Random sequence generation | Low risk | | Permuted block randomization via centralized interactive voice response system or website |
| Allocation concealment | Low risk | | The centralized random permuted block design will be used to balance  treatment assignments across the strata. |
| Blinding of participants and personnel | High risk | | Open label study and patients was orally administered (Crizotinib) or intravenously administered platinumbased chemotherapy (crizotinib). Knowledge of group assignment might have influenced performance. |
| Blinding of outcome assessment | Low risk | | Despite being open label, knowledge of group assignment would not be expected to affect objective outcomes assessment. PFS was assessed by independent review. |
| Incomplete outcome data | Low risk | | Loss to follow-up: chemotherapy 0/171 (4%), crizotinib 2/172 (4%); withdrew consent: chemotherapy 12/172 (5), crizotinib 3/171 (2%); “other reasons”: chemotherapy 4/171 (4%); crizotinib 26/172 (1%). Baseline patient and disease characteristics have been published previously and were similar between treatment arms. |
| Selective reporting | Low risk | | No detected difference in reporting between protocol and publication |
| Other potential threats to validity | Unclear | | Pharmaceutical funding (Pfizer)  Cross-over between treatment groups was permitted; however, steps were taken to mitigate this potential bias: "As prespecified in the protocol, overall survival was also analyzed with the rank-preserving structural failure time model to explore the effect of crossover to crizotinib in the chemotherapy group. All analyses in the chemotherapy group, with the exception of the analysis of overall survival, included only data collected before crossover to crizotinib." |
| **Wu 2018^[3]^ (**PROFILE 1029; **NCT01639001)** | | | |
| Methods: Open-label, randomized, multicenterl, phase 3 study | | | |
| Population: Aged 18-70 years, ALK-positive NSCLC, with no prior systemic treatment, measurable disease according to Response Evaluation criteria in Solid Tumors (RECIST) version 1.1; ECOG PS 0 to 2; adequate hepatic, renal and bone marrow function | | | |
| Interventions: Crizotinib 250 mg BID v. standard platinum-based chemotherapy [Pemetrexed (500 mg/m^2^)/Cisplatin (75 mg/m^2^)Or Pemetrexed(500 mg/m^2^)/Carboplatin (AUC of 5 or 6 mg.min/mL)] | | | |
| Cross-over between treatment groups:Yes: Patients in the chemotherapy arm who had disease progression, as confifirmed by IRR, could cross over to the crizotinib arm if they met the safety screening criteria. | | | |
| Reported outcomes of interest to this review:  PFS (time from randomization to RECIST-defifined progression, as assessed by IRR, or death).  ORR (percentage of patients with a complete or partial response according to RECIST, as assessed by IRR),  Overall survival (OS)  PFS of ALK-positive NSCLC with BM  SAEs (an adverse event that results in death, is life threatening, requires inpatient hospitalization or extends a current hospital stay, results in ongoing or significant incapacity or interferes substantially with normal life functions, or causes a congenital anomaly or birth defect)  AEs leading to discontinuation | | | |
| **Risk of bias** | | | |
| **Bias** | **Judgment** | **Support for judgment** | |
| Random sequence generation | Unclear | Insufficient details to permit judgment | |
| Allocation concealment | Unclear | Insufficient details to permit judgment.  The investigator made the choice of platinum-based chemotherapy. | |
| Blinding of participants and personnel | High risk | Open label study and patients was orally administered (Crizotinib) or intravenously administered platinumbased chemotherapy (crizotinib). Knowledge of group assignment might have influenced performance. | |
| Blinding of outcome assessment | Low risk | Despite being open label, knowledge of group assignment would not be expected to affect objective outcome assessment; PFS, OS and ORR assessed by independent review | |
| Incomplete outcome data | Low risk | One patient was lost-to follow-up in the crizotinib group (of 104), with none lost in the chemotherapy group (of 103); 10 patients in the crizotinib group refused further follow-up, compared with 5 in the chemotherapy group. | |
| Selective reporting | Low risk | No detected selective reporting | |
| Other potential threats to validity | Unclear | Pharmaceutical funding (Pfizer)  80.6% of patients from chemotherapy arm crossed over to the crizotinib arm | |
| Alice T. Shaw2022^[4]^ (CROWN; NCT03052608) | | | |
| **Methods:** Open-label, randomized, global, multi-center, phase 3 study | | | |
| **Population:** patients (≥18 or ≥20 years of age, according to local regulations) had histologically or cytologically confirmed locally advanced or metastatic NSCLC with ALK status determined by means of the Ventana ALK (D5F3) CDx immunohistochemical assay. No previous systemic treatment for metastatic disease was allowed. Patients with asymptomatic treated or untreated CNS metastases were eligible. | | | |
| **Interventions:** oral lorlatinib(100 mg daily) v. oral crizotinib( 250 mg twice daily with each drug to be taken either with or without food) | | | |
| **Cross-over between treatment groups:** Per protocol, crossover between the treatment groups was not permitted. | | | |
| **Reported outcomes of interest to this review:**  PFS:defined as the time from randomization to RECIST-defined disease progression (as determined by blinded independent central review) or death from any cause.  Overall survival  Objective response(ORR)  PFS with brain metastases  SAEs (an adverse event that results in death, is life threatening, requires inpatient hospitalization or extends a current hospital stay, results in ongoing or significant incapacity or interferes substantially with normal life functions, or causes a congenital anomaly or birth defect)  AEs leading to discontinuation | | | |
| **Risk of bias** | | | |
| **Domain** | **Judgment** | **Support for judgment** | |
| Random sequence generation | Low risk | Randomization: Patient number and treatment arm allocation will be operated through an automated system. | |
| Allocation concealment | Low riskr | Randomization: Patient number and treatment arm allocation will be operated through an automated system. | |
| Blinding of participants and personnel | High risk | Blinding was not reported and was likely not done because one treatment was administered orally once daily (lorlatinib) and one was twice daily (crizotinib). Knowledge of group assignment might have influenced performance | |
| Blinding of outcome assessment | High risk | Data were collected by the investigators and analyzed by the sponsor.  PFS based on Blinded Independent Central Review (BICR) assessment (RECIST v.1.1, see Appendix 3). Secondary end points included progression-free survival overall survival, objective response, objective intracranial response, and safety were assessed by the investigator.-改成high | |
| Incomplete outcome data | Low risk | 2 Were lost to follow-up, 22 withdrew consent | |
| Selective reporting | Low risk | No differences in outcomes between the protocol and publication were detected | |
| Other potential threats to validity | Unclear | Pharmaceutical funding (Pfizer) | |
| **Leora Horn 2021^[5]^ (eXalt3; NCT02767804)** | | | |
| **Methods**: Open-label, randomized (1:1), multicenter, global,phase 3 study | | | |
| **Population**: 18 years of age or older, had advanced or recurrent (stage IIIB not amenable formultimodality treatment) or metastatic (stage IV) NSCLC that was ALK positive as determined by local testing, andhadmeasurablediseaseperResponse Evaluation Criteria in Solid Tumours (RECIST), version 1.1. | | | |
| **Interventions**: oral ensartinib(225mg once daily) v. crizotinib(250mg twice daily) | | | |
| **Cross-over between treatment groups:** Crossover was not permitted | | | |
| **Reported outcomes of interest to this review:**  PFS (independent review; progressive disease or death according to RECIST version 1.1)  Overall survival.  ORR  PFS with brain metastases  SAEs (an adverse event that results in death, is life threatening, requires inpatient hospitalization or extends a current hospital stay, results in ongoing or significant incapacity or interferes substantially with normal life functions, or causes a congenital anomaly or birth defect)  AEs leading to discontinuation | | | |
| **Risk of bias** | | | |
| **Domain** | **Judgment** | **Support for judgment** | |
| Random sequence generation | Low risk | centrally via an interactive voice response system using a permuted block randomization was used for sequence generation | |
| Allocation concealment | Low risk | Centralized allocation | |
| Blinding of participants and personnel | High risk | Open label study and one treatment was administered orally once daily (ensartinib) and one was twice daily (crizotinib). Knowledge of group assignment might have influenced performance | |
| Blinding of outcome assessment | Low risk | PFS and overall survival, CNS response rate, and CNS time to progression were  assessed by the blinded independent review committee. | |
| Incomplete outcome data | Low risk | 1 Were lost to follow-up, 9 withdrew consent | |
| Selective reporting | Unclear | No protocol available; | |
| Other potential threats to validity | Unclear | Pharmaceutical funding ( Xcovery Holdings Inc.)  Interim analysis; OS data immature | |
| **Kazuhiko Nakagawa 2020^[6]^ (J-ALEX; JapicCTI-132316; JO28928)** | | | |
| **Methods:** Open-label, multi-site, randomized, phase 3 study (Japan) | | | |
| **Participants:**  20 yr, ALK-positive NSCLC, with ECOG score of 0–2, ALK-inhibitor naive, chemotherapy-naïve or had received 1 regimen of chemotherapy | | | |
| **Interventions:** Alectinib (300 mg BID) vs. Crizotinib (250 mg BID) | | | |
| **Cross-over between treatment groups:** No: Treatment crossover after study withdrawal was allowed in both study groups. | | | |
| **Reported outcomes of interest to this review:**  The primary end point was IRF-assessed PFS.  Secondary end points included investigator-assessed PFS, OS, objective response rate, health-related quality of life, safety (National Cancer Institute Common Terminology Criteria for Adverse Events version 4.0), and pharmacokinetics. | | | |
| **Risk of Bias** | | | |
| **Domain** | **Judgment** | **Quote** | |
| **Bias** | **Judgment** | **Support for judgment** | |
| Random sequence generation | Low risk | Interactive web response system was used for sequence generation | |
| Allocation concealment | Low risk | Interactive web response system was used for allocation concealment | |
| Blinding of participants and personnel | High risk | Open label study in which knowledge of group assignment might have influenced performance. | |
| Blinding of outcome assessment: | Low risk | Despite being open label, knowledge of group assignment would not be expected to affect objective outcomes assessment. Blinded assessment of PFS and OS was defined as the time from randomization to death from any cause. | |
| Incomplete outcome data | Low risk | No patients were lost to follow-up. | |
| Selective reporting | Unclear | Protocol not available. | |
| Other potential threats to validity | Unclear | Pharmaceutical funding (Chugai Pharmaceutical Co.)  Unclear description of crossover between groups: "Treatment crossover after study withdrawal was allowed in both study groups. Patients initially randomly assigned to the crizotinib group who withdrew from the study because of progressive disease before the approval of alectinib (ie, before September, 2014), were permitted to receive alectinib during the study." | |
| T. Mok 2020[^[7]^ **(ALEX; NCT02075840)** | | | |
| **Methods:** Open-label, multi-site, randomized, phase 3 study | | | |
| **Participants:** patients aged ≥18 years with previously untreated stage III/IV ALK-positive NSCLC | | | |
| **Interventions:** Alectinib (600 mg twice daily) v. crizotinib (250 mg twice-daily) | | | |
| **Cross-over between treatment groups:** No: Crossover between treatment arms was not permitted before PD. | | | |
| PFS (investigator assessed [primary]; independent review;  time to disease progression or death whichever occurred first, by use of Response Evaluation Criteria in Solid Tumors (RECIST) Version 1.1 (v1.1) Criteria; PFS was assessed as time to disease progression or death whichever occurred first by investigator assessment using Response Evaluation Criteria in Solid Tumors (RECIST) Version 1.1 (v1.1) Criteria. As per RECIST v1.1, disease progression is a 20% increase in the sum of the diameters of target lesions, an increase in size of measurable lesions by at least 5 millimeter (mm) and the appearance of new lesions.)  overall survival (OS)  objective response rate (ORR)  PFS with brain metastases  SAEs (an adverse event that results in death, is life threatening, requires inpatient hospitalization or extends a current hospital stay, results in ongoing or significant incapacity or interferes substantially with normal life functions, or causes a congenital anomaly or birth defect)  AEs leading to discontinuation | | | |
| **Risk of bias** | | | |
| **Bias** | **Judgment** | **Support for judgment** | |
| Random sequence generation | Low risk | Participants were randomly allocated by use of a block stratified randomization procedure. | |
| Allocation concealment | Low risk | Allocation was concealed by use of an interactive or Web-based system | |
| Blinding of participants and personnel | High risk | Open label study in which knowledge of group assignment might have influenced performance | |
| Blinding of outcome assessment | Low risk | Despite being open label, knowledge of group assignment would not be expected to affect objective outcomes assessment. The primary outcome (PFS) was based on unblinded investigator’s assessment; secondary analysis using independent assessment; however, the two estimates were similar: (Investigator HR: 0.47 [95% CI, 0.34 to 0.65]; independent review HR: 0.50 [95% CI, 0.36 to 0.70] | |
| Incomplete outcome data | High risk | Lost to follow-up or “declined to participate”: alectinib 17/152 (10%), crizotinib 27/151 (18%); discontinued treatment due to “other reasons”: alectinib 3/152 (2%), crizotinib 2/151 (1%); unbalanced discontinuations and withdrawals between groups could have affected the direction and magnitude of the effect size | |
| Selective reporting | Low risk | No differences in outcomes between the protocol and publication were detected | |
| Other potential threats to validity | Unclear risk | Pharmaceutical funding (Hoffmann–La Roche) | |
| **Shaw 2017^[8]^ (ASCEND-5, NCT01828112)^8,14^** | | | |
| **Methods**: Open-label, multisite, randomized, phase 3 trial | | | |
| **Population**:  18 yr, ALK-positive NSCLC, with WHO performance status of 0–2, one or two previous chemotherapy regimens and previous crizotinib for at least 21 d | | | |
| **Interventions**: Ceritinib (750 mg per day, fasted, in continuous 21 day treatment cycles) v. chemotherapy (intravenous pemetrexed 500 mg/m² or docetaxel 75 mg/m² [investigator choice], every 21 days). | | | |
| **Cross-over between treatment groups:** Yes, Patients randomly assigned to chemotherapy were allowed to crossover to ceritinib. | | | |
| **Reported outcomes of interest to this review:**  Progression-free survival (the time from randomisation to the first radiologically documented disease progression [according to RECIST 1.1 and assessed by the masked IRC] or death from any cause).  Overall survival (the time from randomisation to death from any cause).  Overall response (the proportion of patients with a best overall response of complete response or partial response)  PFS with brain metastases  SAEs (an adverse event that results in death, is life threatening, requires inpatient hospitalization or extends a current hospital stay, results in ongoing or significant incapacity or interferes substantially with normal life functions, or causes a congenital anomaly or birth defect).  AEs leading to discontinuation | | | |
| **Risk of bias** | | | |
| **Domain** | **Judgment** | **Support for judgment** | |
| Random sequence generation | Low risk | Randomly allocated eligible patients using interactive response technology (IRT) in a 1:1 ratio (block randomisation with a block size of four used) | |
| Allocation concealment | Low risk | A separate medication randomisation list was produced under the responsibility of Novartis using a validated system that automated the random assignment of medication numbers to medication packs containing ceritinib. Chemotherapy was locally sourced and the medication number was provided by the IRT system | |
| Blinding of participants and personnel | High risk | Open label study in which knowledge of group assignment might have affected subjective outcome assessment, e.g., treatment-related death that involves judgment on the causal relationship between drug and death. | |
| Blinding of outcome assessment | Low risk | Despite being open label, knowledge of group assignment would not be expected to affect objective outcomes assessment. Outcomes were assessed both locally by investigators and by a masked independent review committee | |
| Incomplete outcome data | Low risk | No patients were lost to follow-up. Withdraw due to patient or guardian decision: chemotherapy 7/105 (7%), ceritinib 6/82 (7%). Withdrawal due to physician decision: chemotherapy 3/105 (3%), ceritinib 5/82 (6%). Given the low and balanced discontinuations and withdrawals between groups, attrition bias is estimated to be modest. | |
| Selective reporting | Low risk | No detected difference in reporting between protocol and publication | |
| Other potential threats to validity | Unclear | Pharmaceutical funding (Novartis)  Cross-over to ceritinib permitted during the extension; authors note that differences in overall survival were “probably confounded by the high proportion of cross-over of patients from the chemotherapy group to the ceritinib group” | |
| **Soria 2017^[9]^ (ASCEND-4; NCT01828099)** | | | |
| **Methods:** Open-label, randomized, multi-national, phase 3 study | | | |
| **Population:**  18 yr, ALK-positive NSCLC, ECOG score of 0–2, previously untreated | | | |
| **Interventions:** Ceritinib 750 mg/d v. intravenous chemotherapy (cisplatin [75 mg/m²], or carboplatin [target area under the curve of 5–6] plus pemetrexed [500 mg/m²]) given every 21 days | | | |
| **Cross-over between treatment groups**:Yes, Patients randomly assigned to chemotherapy were allowed to crossover to ceritinib if they had blinded independent review committee confirmed RECIST-defined progressive disease. | | | |
| **Reported outcomes of interest to this review:**  Progression-free survival, defined as the time from randomisation to the date of the first radiologically documented disease progression (assessed by the blinded independent review committee according to RECIST 1.1) or death due to any cause.  Overall survival.  Overall response rate by the blinded independent review committee and the investigator;  PFS with brain metastases  SAEs (an adverse event that results in death, is life threatening, requires inpatient hospitalization or extends a current hospital stay, results in ongoing or significant incapacity or interferes substantially with normal life functions, or causes a congenital anomaly or birth defect)  AEs leading to discontinuation | | | |
| **Bias** | **Judgment** | **Support for judgment** | |
| Random sequence generation | Low risk | Patients were randomized via use of “Interactive Response Technology” (includes Interactive Voice Response System and Interactive Web Response System) | |
| Allocation concealment | Low risk | Centralized allocation | |
| Blinding of participants and personnel | High risk | Open label study in which knowledge of group assignment might have influenced performance | |
| Blinding of outcome assessment | Low risk | Despite being open label, knowledge of group assignment would not be expected to affect objective outcomes assessment | |
| Incomplete outcome data | Low risk | Withdrawals due to “other” reasons: chemotherapy 9/187 (5%), ceritinib 12/189 (6%);Given the low and balanced discontinuations and withdrawals between groups, the risk of attrition bias is estimated to be modest. | |
| Selective reporting | Low risk | No detected difference in reporting between protocol and publication | |
| Other potential threats to validity | Unclear | Pharmaceutical funding (Novartis)  Cross-over between treatment groups allowed | |
| D. Ross Camidge 2021^[10]^ **(ALTA-1L; NCT02737501)** | | | |
| **Methods:** Open-label, randomized, international multicenter, phase 3 study | | | |
| **Population:** ≥18 yr, ALK-positive locally advanced or metastatic NSCLC with at least one measurable lesion according to the Response Evaluation Criteria in Solid Tumors (RECIST), version 1; had not previously received an ALK-targeted therapy | | | |
| **Interventions:** Oral brigatinib (180 mg once daily after a 7-day lead-in period of 90 mg once daily) v. oral crizotinib 250 mg twice daily | | | |
| **Cross-over between treatment groups:** Yes: “Crossover from crizotinib to brigatinib was offered after BIRC-assessed progression (following ≥10-day washout from crizotinib).” | | | |
| **Reported outcomes of interest to this review:**  PFS (independent review committee (IRC), per RECIST v1.1; time interval from the date of the first dose of the study treatment until the first date at which disease progression is objectively documented, or death due to any cause, whichever occurs first.  Objective response rate (ORR) was assessed by BIRC  PFS with brain metastases  overall survival (OS)  SAEs (an adverse event that results in death, is life threatening, requires inpatient hospitalization or extends a current hospital stay, results in ongoing or significant incapacity or interferes substantially with normal life functions, or causes a congenital anomaly or birth defect)  AEs leading to discontinuation | | | |
| **Risk of bias** | | | |
| **Domain** | **Judgment** | **Support for judgment** | |
| Random sequence generation | Unclear | Specific instructions for randomization will be supplied in the Study Reference Manual. No Study Reference Manual available; | |
| Allocation concealment | Unclear | Allocation concealment not described | |
| Blinding of participants and personnel | High risk | This study is unblinded; patients, investigators, and the sponsor will know the identity of each patient’s study drug. Open label study in which knowledge of group assignment might have influenced performance | |
| Blinding of outcome assessment: | Low risk | An independent Data Monitoring Committee (DMC), consisting of 3 to 5 members not associated with the conduct of the study A central blinded Independent Review Committee (BIRC) will evaluate all images collected during the study for the primary endpoint of PFS as well as several secondary endpoints. | |
| Incomplete outcome data | Low risk | More discontinuations occurred in the crizotinib group mainly because of disease progression; about half crossed over to the other arm; however, PFS was assessed before cross-over; interim analysis was appropriate with alpha spending functions. | |
| Selective reporting | High risk | Published protocol includes a section titled "exploratory endpoints" but the information has been redacted. | |
| Other potential threats to validity | Unclear | Pharmaceutical funding (Ariad Pharmaceuticals) Cross-over between treatment groups allowed | |
| **Novello 2018^[11]^ (ALUR; NCT02604342)** | | | |
| **Methods:** Open-label, randomized, multi-national, phase 3 study | | | |
| **Population:** histologically/cytologically confirmed advanced, recurrent, or metastatic ALK-positive NSCLC; two prior lines of systemic therapy (including one line of PDC and one of crizotinib); measurable disease (Response Evaluation Criteria in Solid Tumors [RECIST] v1.1); Eastern Cooperative Oncology Group performance status (ECOG PS) 0–2. | | | |
| **Interventions:** Alectinib 600 mg twice daily v. chemotherapy (pemetrexed 500 mg/m2 or docetaxel 75 mg/m2, every 3 weeks, at the investigators’ discretion) | | | |
| **Cross-over between treatment groups:** Yes: “Crossover from chemotherapy to alectinib was permitted following  progression.” | | | |
| **Reported outcomes of interest to this review:**  OS  PFS (investigator-assessed [primary]; secondary analysis included independent assessment; time from randomization to the first documented disease progression, as determined using RECIST v1.1, or death from any cause, whichever occurred first. As per RECIST v1.1, disease progression is a 20% increase in the sum of the diameters of target lesions, an increase in size of measurable lesions by at least 5 millimeter (mm) and the appearance of new lesions)  PFS of ALK-positive NSCLC with BM  ORR  SAEs (an adverse event that results in death, is life threatening, requires inpatient hospitalization or extends a current hospital stay, results in ongoing or significant incapacity or interferes substantially with normal life functions, or causes a congenital anomaly or birth defect)  AEs leading to discontinuation | | | |
| **Risk of bias** | | | |
| **Domain** | **Judgment** | **Support for judgment** | |
| Random sequence generation | Low risk | a block-stratified randomization procedure (block size 6) using an interactive voice or web-based response system using the following stratification factors: ECOG PS (0/1 versus 2) | |
| Allocation concealment | Low risk | an interactive voice or web-based response system | |
| Blinding of participants and personnel | High risk | Open label study in which knowledge of group assignment might have influenced performance | |
| Blinding of outcome assessment | Low risk | Despite being open label, knowledge of group assignment would not be expected to affect objective outcomes assessment. The Sponsor and the study team performing the primary analysis were blinded to randomized treatment assignments until after database lock. | |
| Incomplete outcome data | Low risk | The ITT population comprised all patients randomized. | |
| Selective reporting | Low risk | Data for outcomes listed in NCT record have been reported in the full publication or in the NCT record | |
| Other potential threats to validity | Unclear | Pharmaceutical funding (Hoffmann-La Roche) Cross-over between treatment groups allowed | |
| **Zhou 2019^[12]^ (ALESIA; NCT02838420)** | | | |
| **Methods:** Open-label, randomized, multi-national, phase 3 study | | | |
| **Population:**  18 yr Asian patients with histologically or cytologically confirmed stage 3b or 4 ALK-positive NSCLC, had not received previous systemic therapy for advanced NSCLC, had measurable disease at baseline (according to Response Evaluation Criteria in Solid Tumours [RECIST] version 1.1), an Eastern Cooperative Oncology Group performance status (ECOG PS) of 0–2, and a life expectancy of at least 12 weeks. | | | |
| **Interventions:** Alectinib (600 mg twice per day; oral) v. crizotinib (250 mg twice per day; oral with or without food) | | | |
| **Cross-over between treatment groups:** No: “Crossover between study groups was not permitted” | | | |
| **Reported outcomes of interest to this review:**  PFS (investigator assessed; time (in months) from randomization to the first documentation of disease progression, as determined by the investigators, or to death from any cause, whichever occurred first; Determined by Investigator Using Response Evaluation Criteria in Solid Tumor (RECIST) v1.1)  OS  Objective response rate  PFS of ALK-positive NSCLC with BM  SAEs (an adverse event that results in death, is life threatening, requires inpatient hospitalization or extends a current hospital stay, results in ongoing or significant incapacity or interferes substantially with normal life functions, or causes a congenital anomaly or birth defect)  AEs leading to discontinuation | | | |
| **Risk of bias** | | | |
| **Domain** | **Judgment** | **Support for judgment** | |
| Random sequence generation | Low risk | "Randomisation was done centrally via an interactive voice or web response system" "Patients were randomly assigned via a block-stratified (block size three) randomisation procedure in a 2:1 ratio." | |
| Allocation concealment | Low risk | Randomization was done centrally via an interactive voice or web response system | |
| Blinding of participants and personnel | Low risk | Clinical staff involved in the study at investigative sites and the funder’s drug safety and medical monitoring staff had access to information outlining the treatments assigned to individual patients during the study to monitor safety and to do routine data cleaning activities. However, the independent review committee remained masked to treatment assignment, and funder personnel did not have access to efficacy and safety summaries by treatment group, before the formal reporting of study results | |
| Blinding of outcome assessment | Low risk | independent review committee-assessed the outcome.  The differences in effect estimate for investigator assessment PFS (HR 0.22, 95% CI 0.13–0.38) and independent assessment PFS (HR 0.37, 95% CI 0.22–0.61) weren’t noted. | |
| Incomplete outcome data | Low risk | Discontinuations similar across groups for the discontinued treatment due to withdrew consent (1 vs. 1); primary analysis based on ITT; no cross-over allowed | |
| Selective reporting | High risk | NCT record lists 12 outcomes and but data not reported for all outcomes (not stated why data reported for some but not all outcomes) | |
| Other potential threats to validity | Unclear | Pharmaceutical funding (Hoffmann-La Roche): “The funder was involved in the study design, data collection, data analysis, data interpretation, and writing of the Article.” | |
| Note: OS = overall survival, PFS = progression-free survival, ORR=Objective response rate, SAEs= Serious adverse events, AEs= Adverse events | | | |

**Figure S1: Convergence of the three chains established by inspection of the history feature and the Brooks-Gelman-Rubin diagnostic**


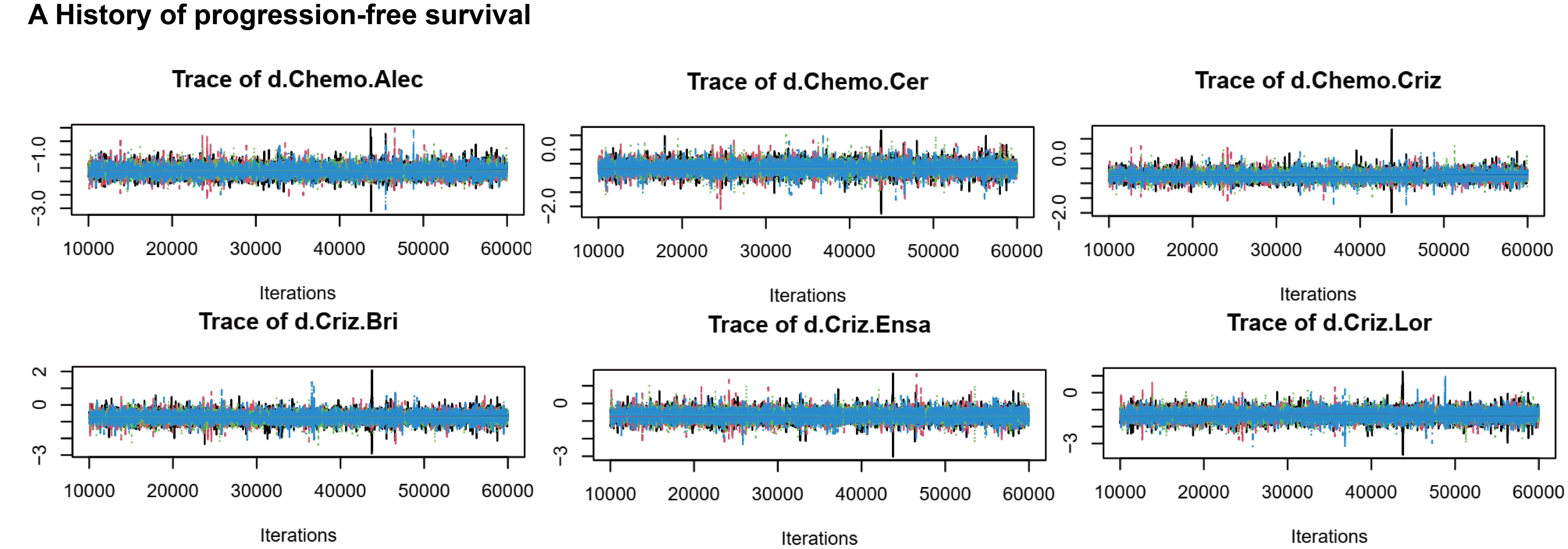


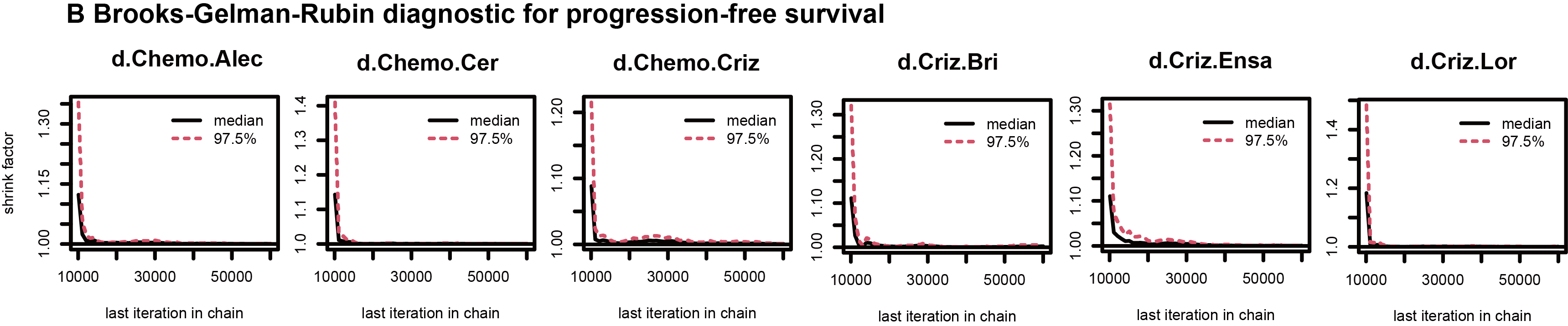


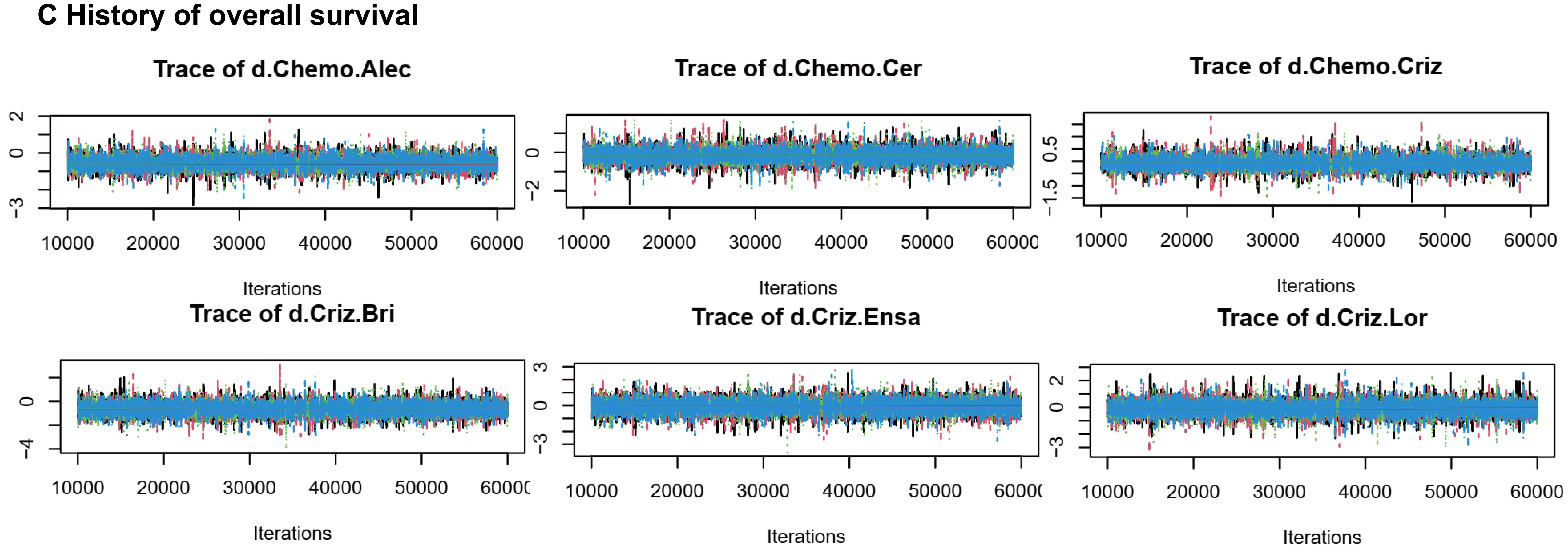


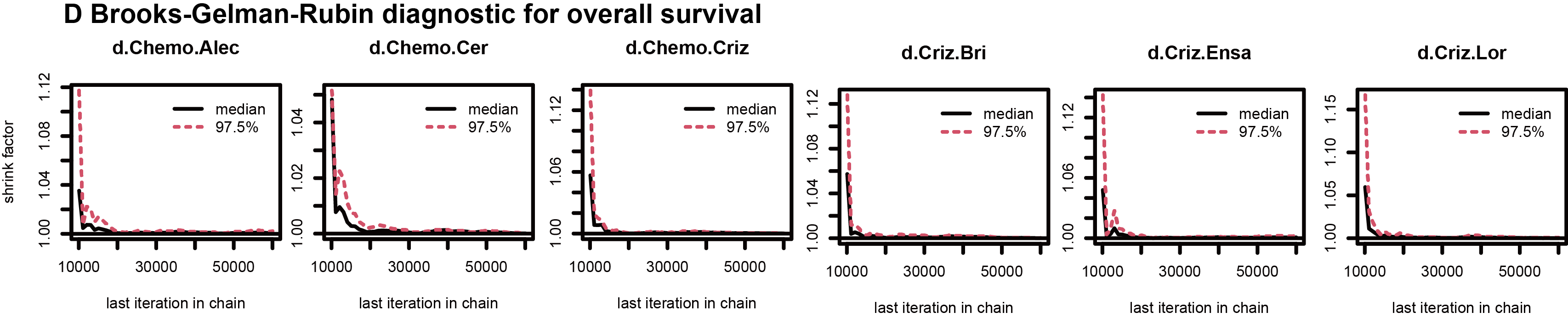


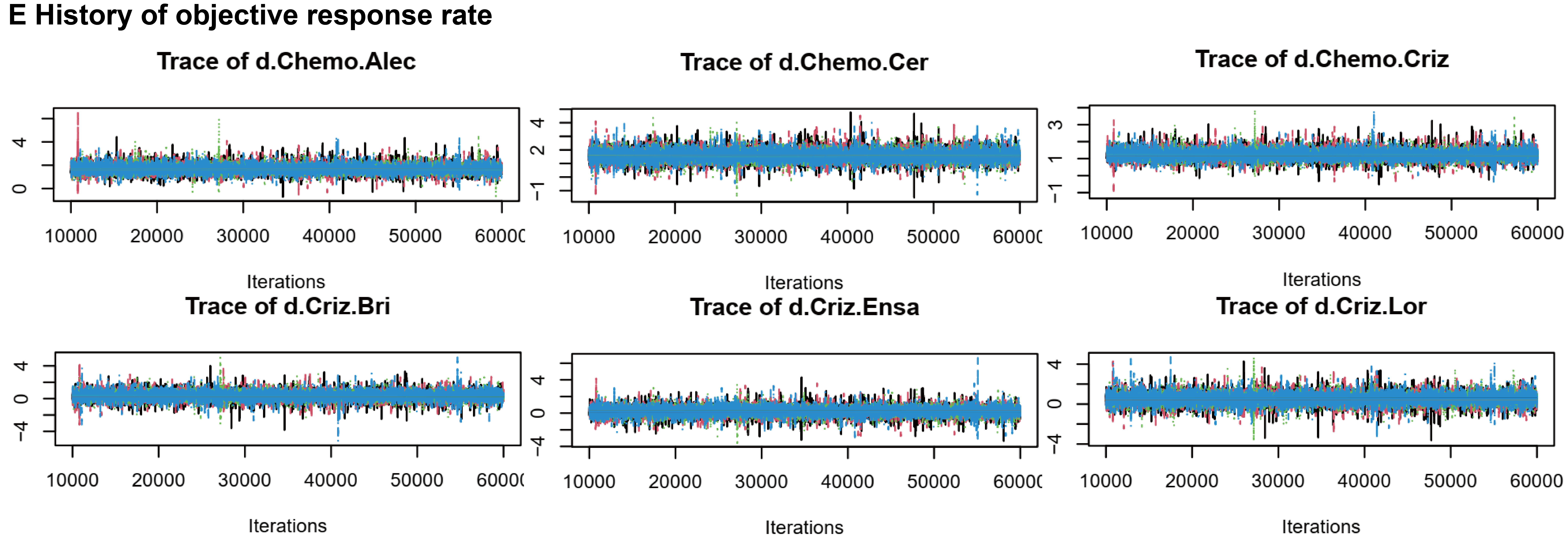


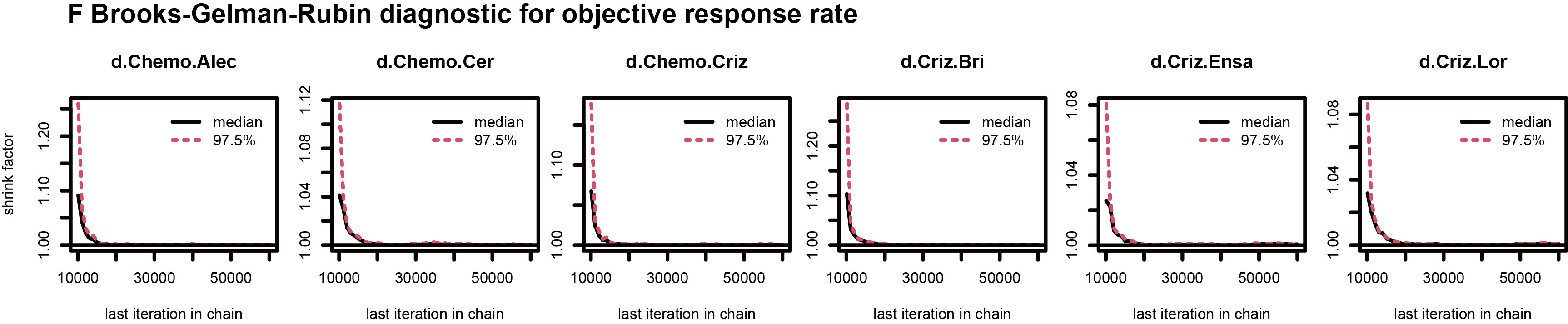


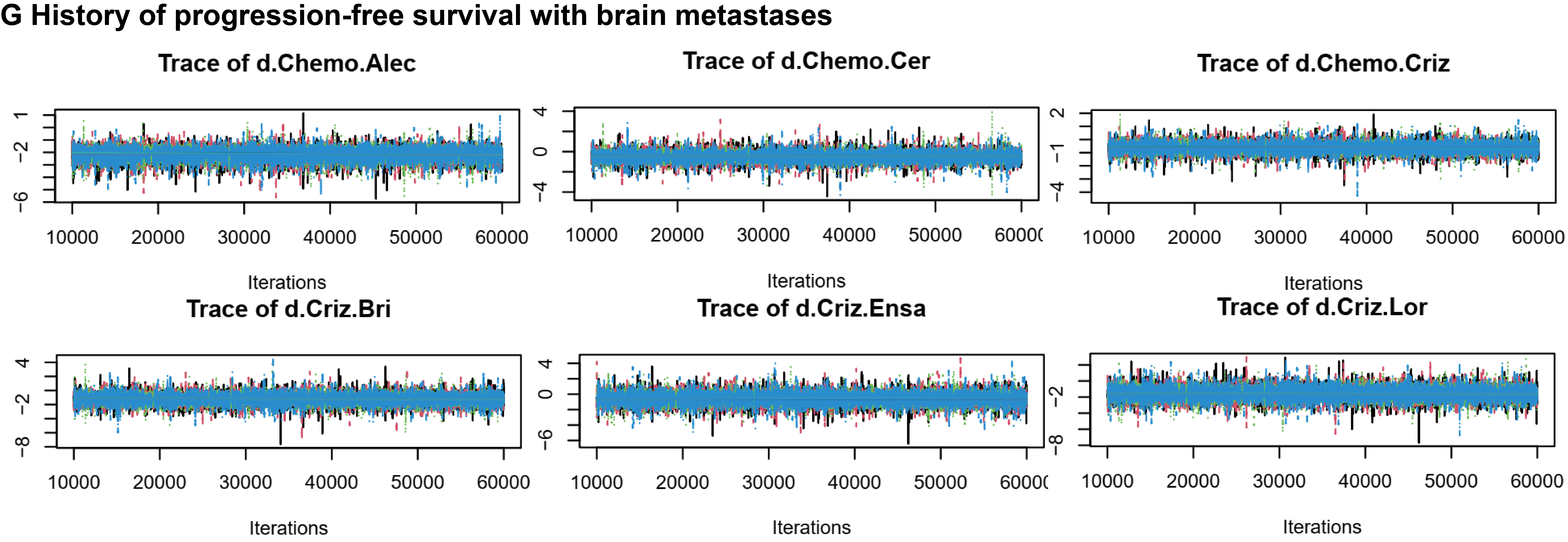


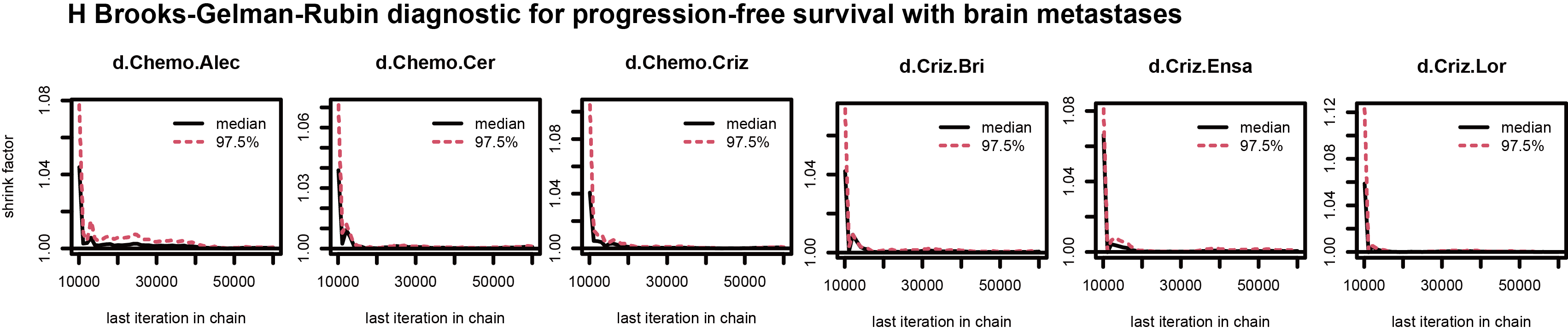


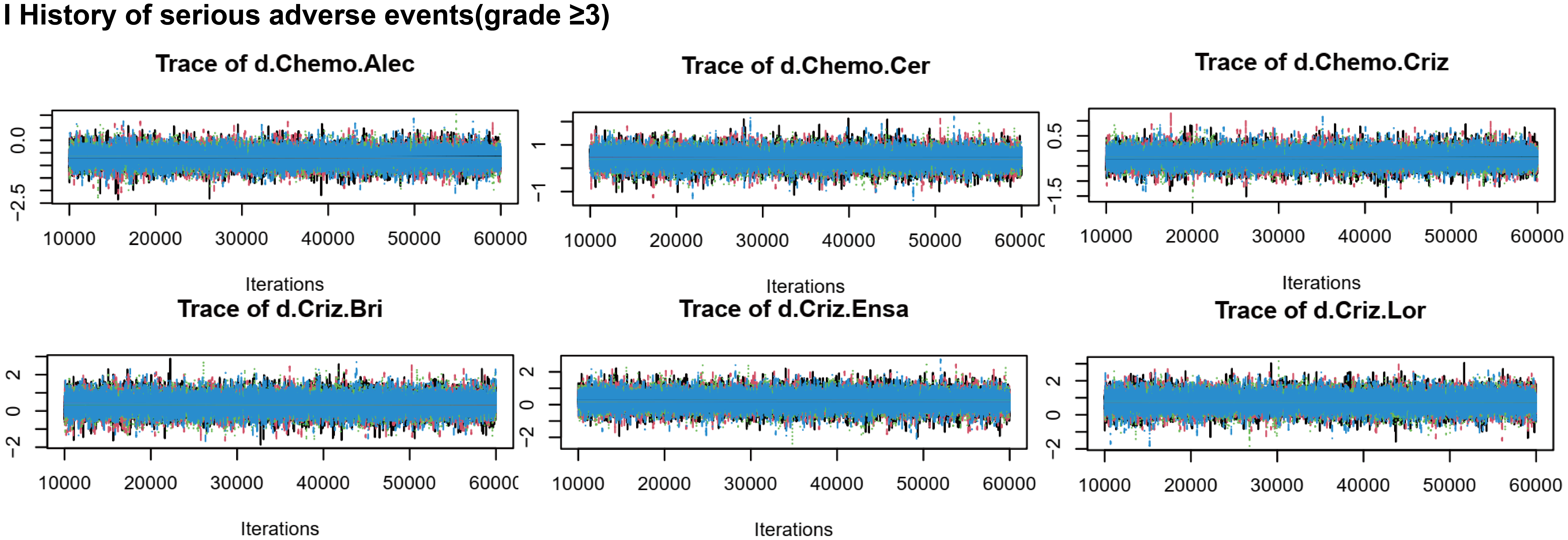


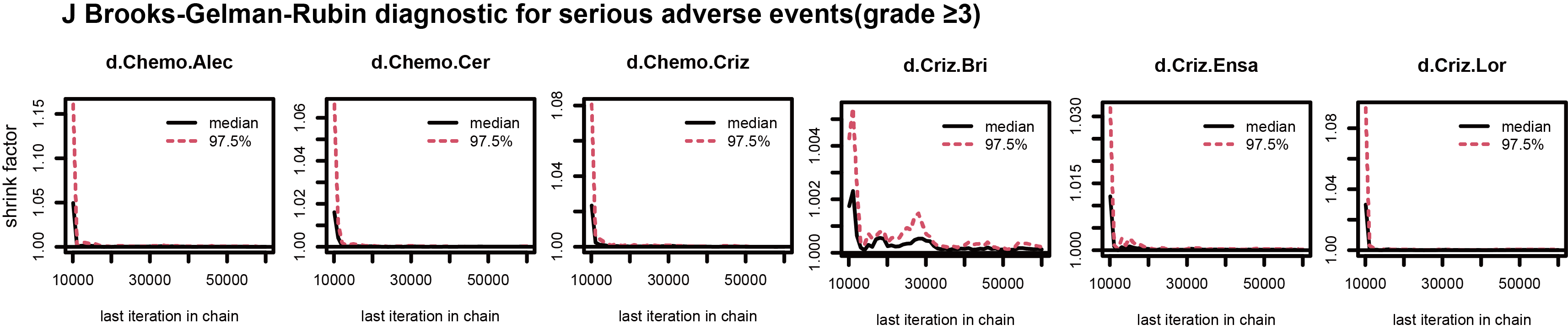


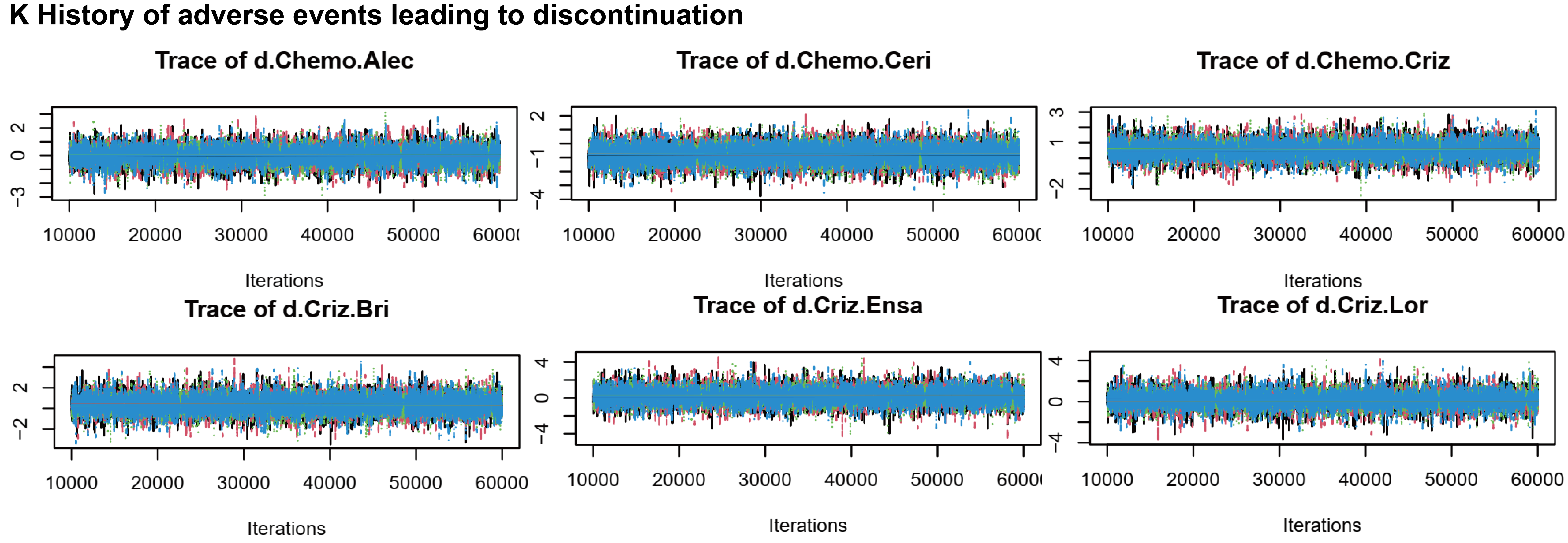


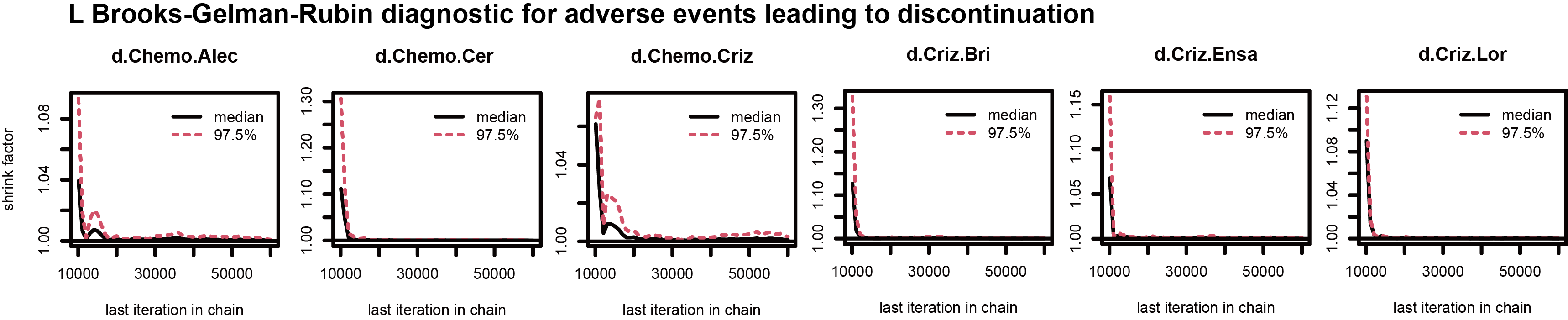


Convergence of the three chains established by inspection of the history feature and the BrooksGelman-Rubin diagnostic for progression-free survival (A and B), overall survival (C and D), objective response rate (E and F), progression-free survival with brain metastases (G and H), grade ≥3 adverse events (I and J) and adverse events leading to discontinuation (K and L).

Chemo: chemotherapy, Alec: alectinib, Cer: ceritinib, Criz: crizotinib, Bri: brigatinib, Ensa: ensartinib, Lor: lorlatinib.

**Figure S2: Bland Altman plot**


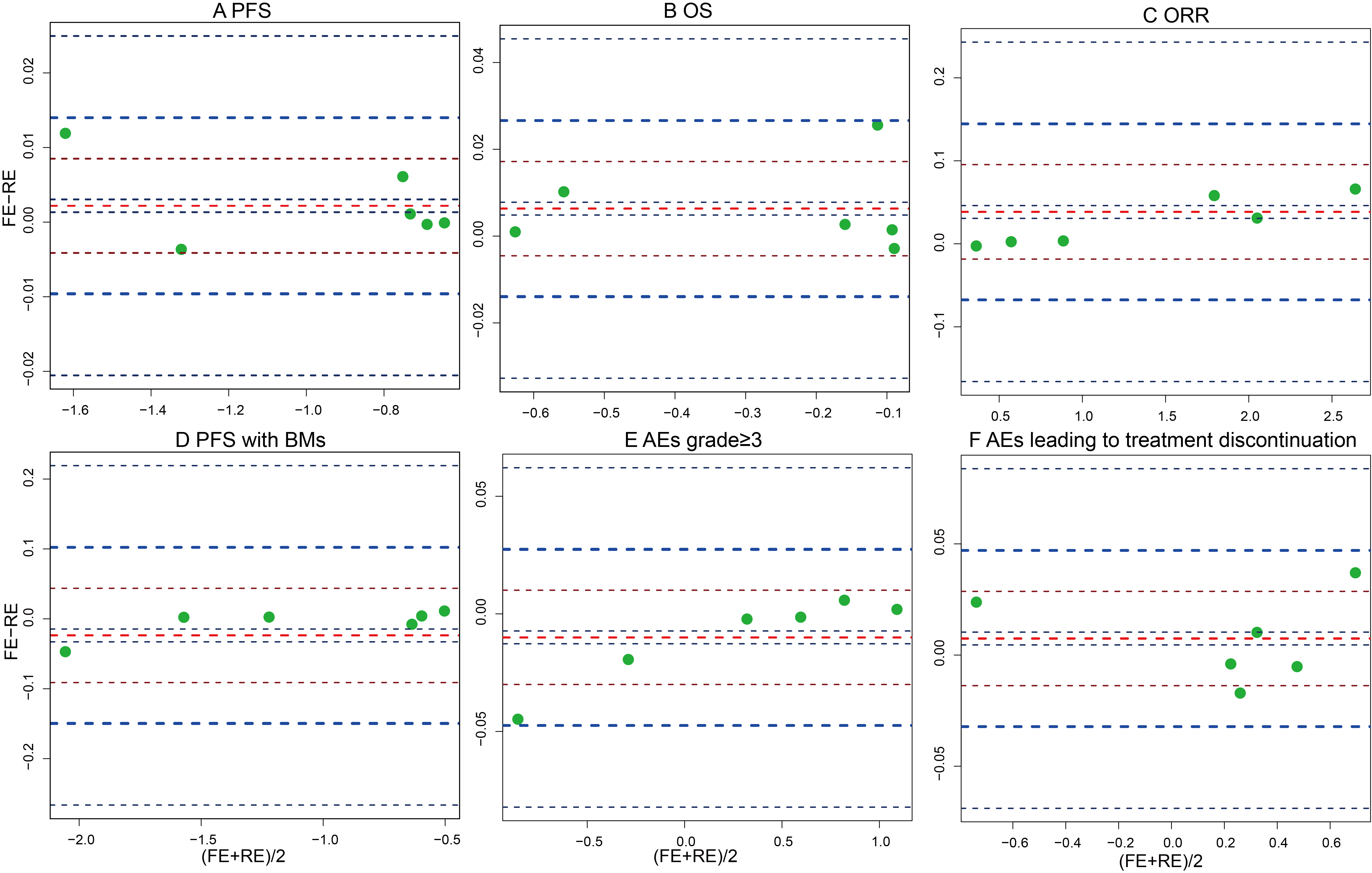


Bland Altman plot for progression-free survival (A ); overall survival(B); objective response rate(C); progression-free survival with brain metastases(D); grade≥3 adverse events(E); and adverse events leading to discontinuation (F).

**Figure S3:** **Forest plots depicting results of head-to-head comparisons according to pairwise meta-analyses**


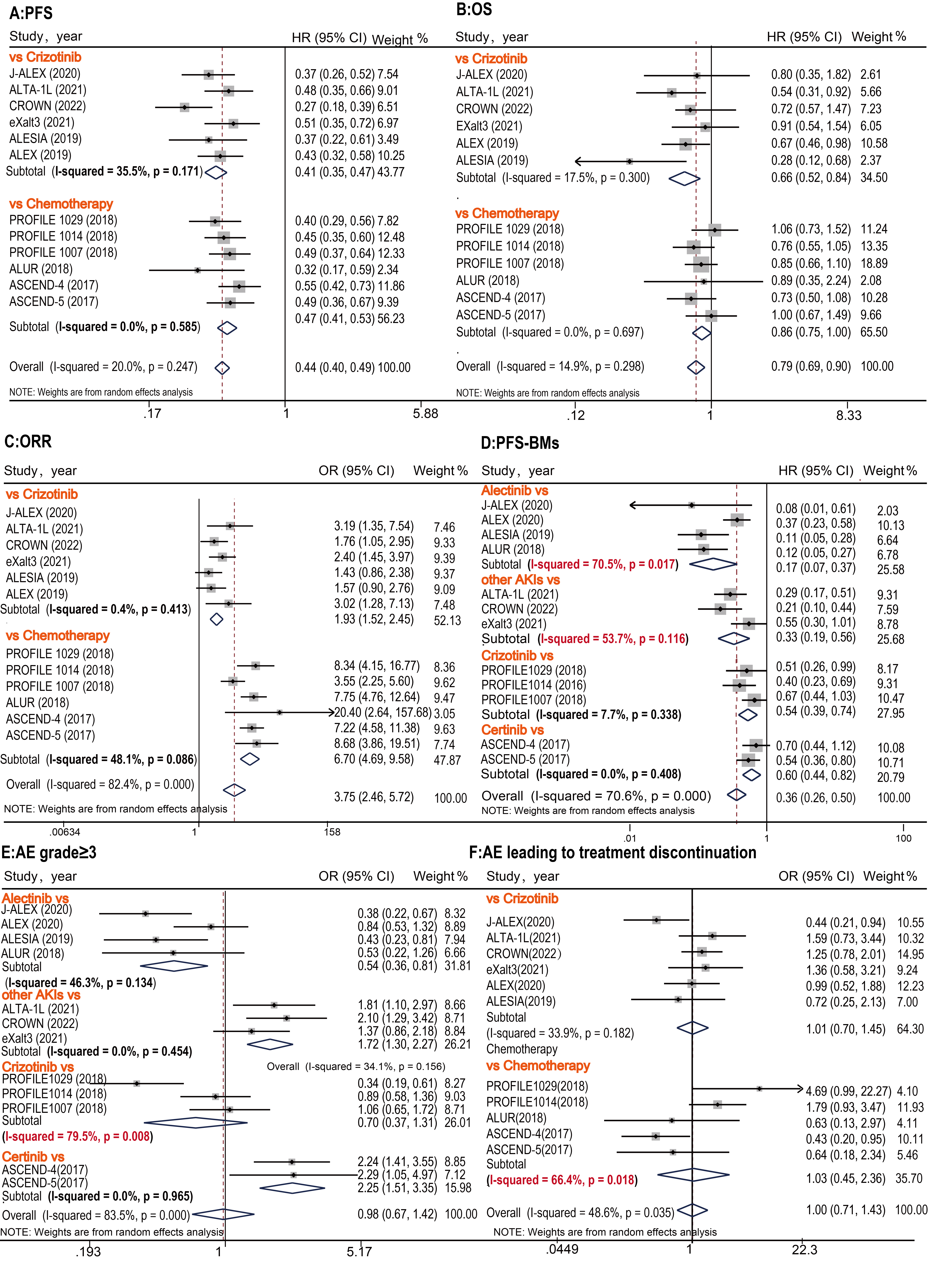


Forest plots depicting results of head-to-head comparisons according to pairwise meta-analyses on different outcomes in ALK-positive NSCLC patients (A) PFS, Progression-free survival; ; (B) OS,Overall survival;(C) ORR, Objective response rate; (D) PFS-BM, Progression-free Survival with brain metastases; (E) AE grade≥3, grade≥3 adverse events; (F) Adverse events leading to discontinuation. Results of heterogeneity assessments are adherently presented. HR=hazard ratio; OR=odds ratio; CI=confidence interval.

**Figure S4: Forest plots depicting results of head-to-head comparisons in according to pairwise and network meta-analyses.**


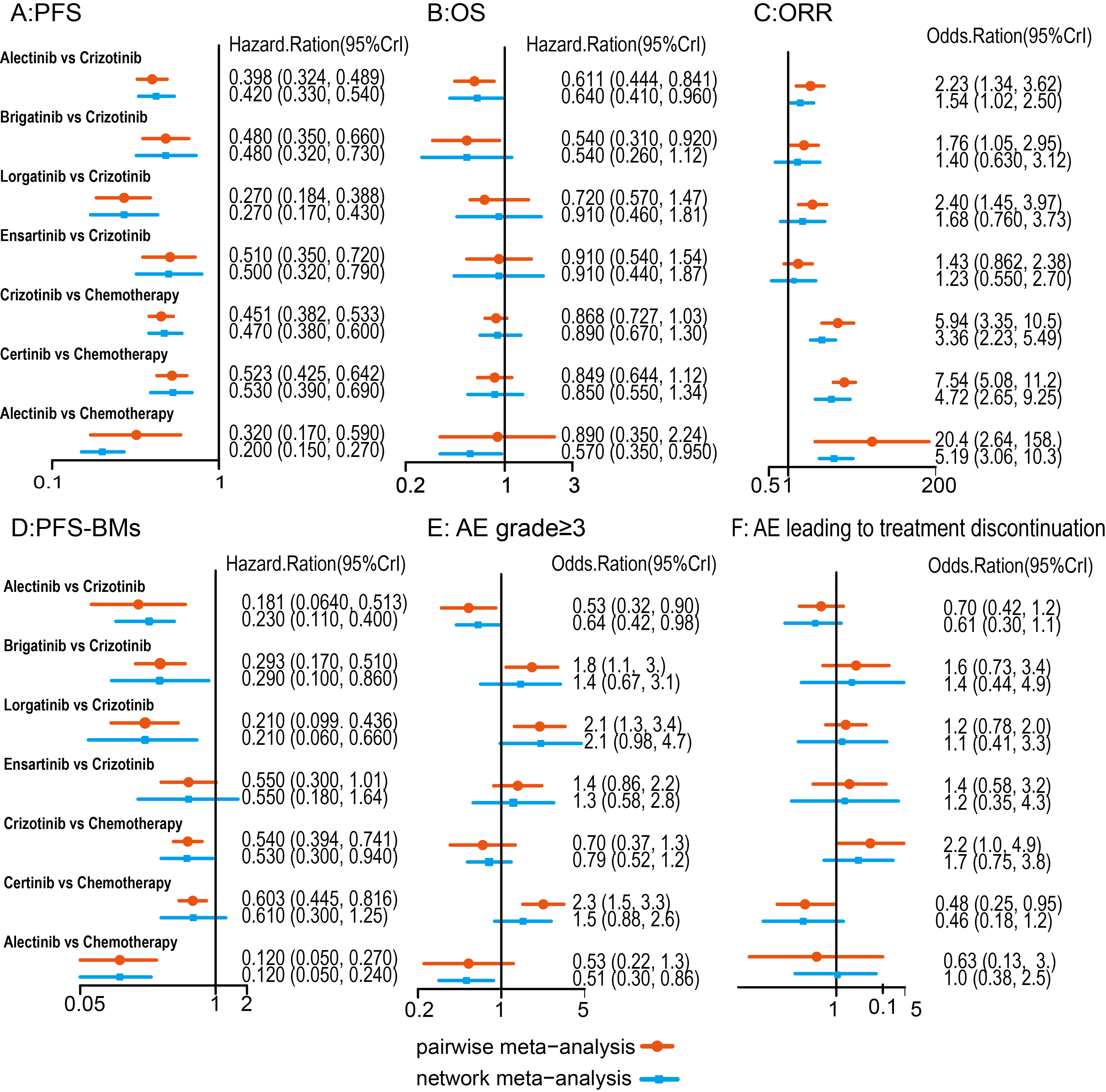


Forest plots depicting results of head-to-head comparisons in according to pairwise and network meta-analyses. Results of all comparisons in ALK-positive NSCLC, and brain metastases subpopulations were consistent between pairwise meta-analysis (orange) and network meta-analysis (blue). (A) PFS, Progression-free survival;; (B) OS,Overall survival;(C) ORR, Objective response rate; (D) PFS-BM, Progression-free Survival with brain metastases; (E) AE grade≥3; (F) AE leading to discontinuation. CrI=credible interval.

**Figure S5:Node-splitting analysis of inconsistency.**


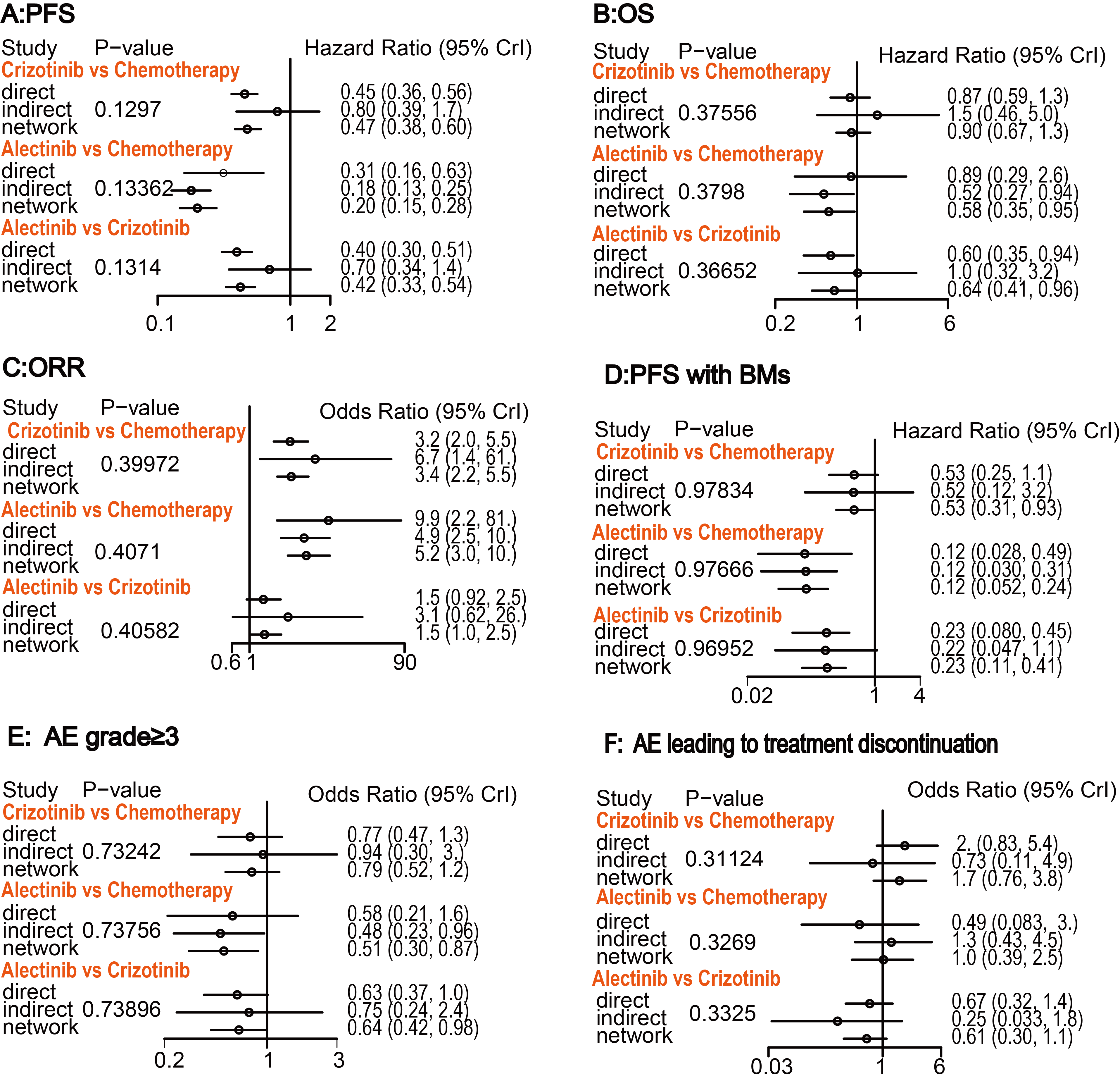


No significant values (P ≤ 0.05) are found, indicating no significant inconsistency between the direct effect and indirect effects. (A) PFS, Progression-free survival;; (B) OS,Overall survival;(C) ORR, Objective response rate; (D) PFS-BM, Progression-free Survival with brain metastases; (E) AE grade≥3; (F) AE leading to discontinuation. CrI=credible interval.

**Figure S6: Comparison-adjusted funnel plots for the primary endpoints and adverse events.**


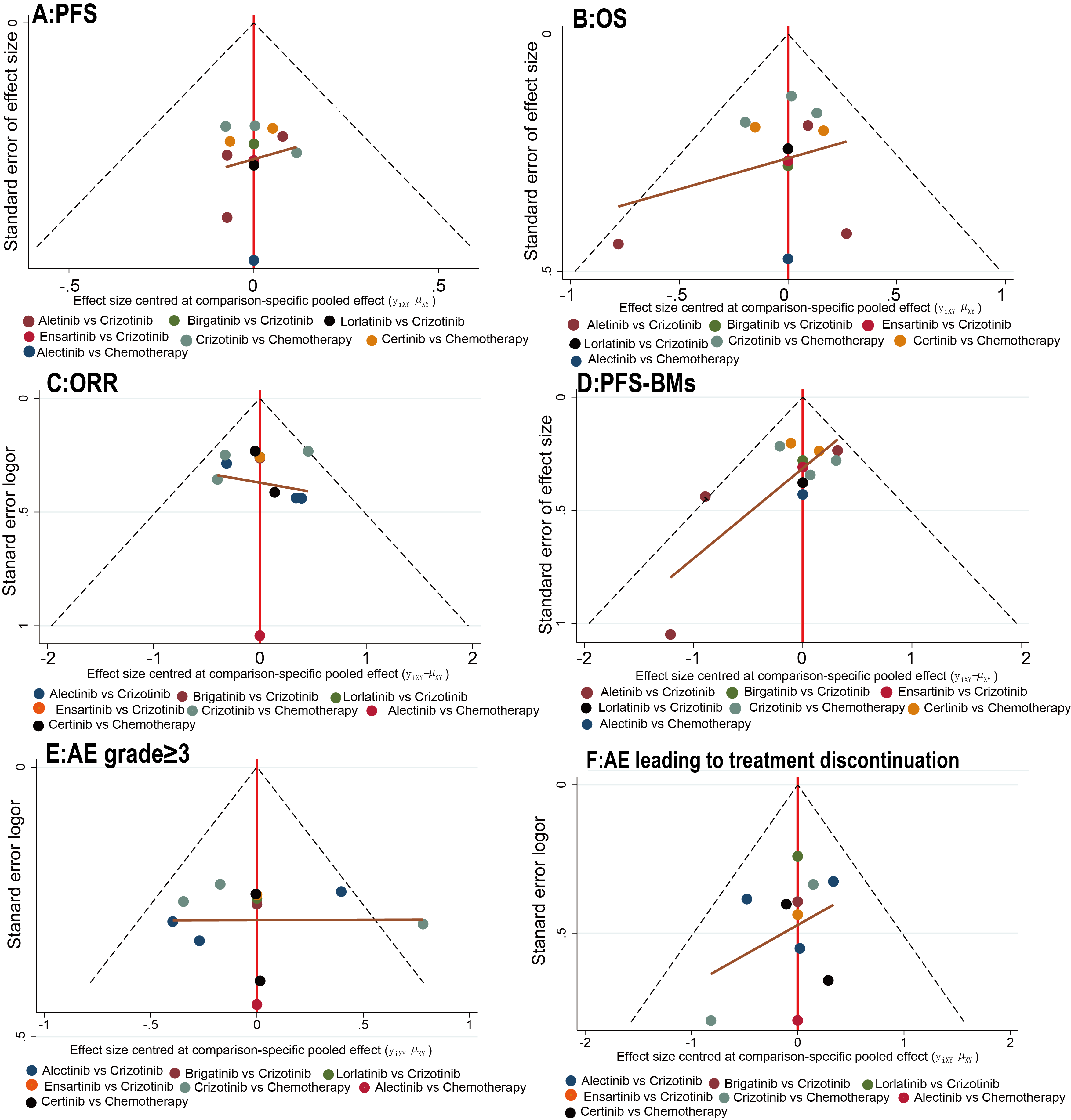


(A) PFS: Progression-free survival; (B) OS: Overallsurvival; (C) ORR: Objective response rate; (D) PFS-BM: Progression-free Survival with brain metastases; (E) Adverse events grade ≥3; (F) Adverse event leading to treatment discontinuation.

**Table S1. Bayesian ranking results and SUCRA of network meta-analysis.**

| Treatment | Rank of possibility (%) | | | | | | | SUCRA |
| --- | --- | --- | --- | --- | --- | --- | --- | --- |
|  | 1 | 2 | 3 | 4 | 5 | 6 | 7 |  |
| Progression-free survival | | | | | | | | |
| Alectinib | 2.87 | 56.20 | 31.53 | 9.35 | 0.04 | 0.00 | 0.00 | 75.41 |
| Brigatinib | 2.37 | 21.29 | 36.78 | 38.97 | 0.43 | 0.013 | 0.03 | 64.29 |
| Ceritinib | 0.00 | 0.04 | 0.17 | 0.99 | 25.18 | 73.52 | 0.09 | 21.30 |
| Crizotinib | 0.00 | 0.00 | 0.02 | 0.42 | 73.59 | 25.95 | 0.02 | 29.08 |
| Lorlatinib | **92.74** | 5.07 | 1.62 | 0.52 | 0.04 | 0.00 | 0.00 | 98.32 |
| Ensartinib | 2.01 | 17.40 | 29.88 | 49.74 | 0.71 | 0.23 | 0.03 | 61.57 |
| Chemotherapy | 0.00 | 0.00 | 0.00 | 0.00 | 0.00 | 0.16 | **99.83** | 0.03 |
| Overall survival | | | | | | | |  |
| Alectinib | 25.27 | 47.61 | 18.59 | 5.92 | 1.68 | 0.65 | 0.29 | 80.96 |
| Brigatinib | **60.57** | 20.98 | 9.41 | 4.06 | 1.95 | 1.52 | 1.52 | 87.25 |
| Ceritinib | 2.43 | 6.86 | 18.78 | 22.43 | 19.22 | 17.71 | 12.56 | 41.25 |
| Crizotinib | 0.01 | 0.39 | 6.59 | 24.79 | 37.74 | 24.77 | 6.08 | 33.71 |
| Lorlatinib | 5.51 | 11.46 | 22.30 | 18.14 | 12.03 | 11.29 | 19.28 | 44.88 |
| Ensartinib | 6.14 | 12.18 | 21.91 | 17.23 | 11.22 | 10.74 | 20.60 | 45.03 |
| Chemotherapy | 0.007 | 0.53 | 2.43 | 7.43 | 16.54 | 33.32 | **39.68** | 16.92 |
| Objective response rate | | | | | | | | |
| Alectinib | 17.78 | 30.29 | 28.23 | 16.89 | 5.88 | 0.92 | 0.01 | 72.40 |
| Brigatinib | 16.84 | 18.01 | 19.11 | 19.99 | 14.99 | 10.67 | 0.30 | 61.43 |
| Ceritinib | 17.09 | 18.07 | 19.52 | 19.75 | 14.77 | 10.75 | 0.06 | 61.75 |
| Crizotinib | 0.01 | 0.27 | 2.30 | 10.93 | 34.00 | 52.25 | 0.00 | 27.32 |
| Lorlatinib | **39.63** | 21.62 | 15.78 | 11.76 | 6.80 | 4.21 | 0.20 | 77.01 |
| Ensartinib | 8.66 | 11.65 | 15.07 | 20.66 | 23.43 | 20.03 | 0.51 | 49.89 |
| Chemotherapy | 0.00 | 0.00 | 0.00 | 0.03 | 0.12 | 0.94 | **98.91** | 0.21 |
| PFS with BM | | | | | | | | |
| Alectinib | 31.88 | 43.70 | 22.03 | 2.28 | 0.08 | 0.01 | 0.00 | 84.16 |
| Brigatinib | 16.44 | 25,76 | 42.80 | 12.50 | 1.47 | 0.60 | 0.43 | 73.28 |
| Ceritinib | 0.11 | 0.39 | 1.62 | 8.38 | 25.92 | 57.79 | 5.79 | 23.97 |
| Crizotinib | 0.00 | 0.03 | 0.38 | 7.54 | 62.28 | 28.64 | 1.14 | 29.58 |
| Lorlatinib | **49.48** | 25.24 | 18.74 | 5.30 | 0.72 | 0.31 | 0.22 | 85.95 |
| Ensartinib | 2.09 | 4.88 | 14.37 | 63.73 | 8.30 | 4.53 | 2.10 | 51.12 |
| Chemotherapy | 0.00 | 0.00 | 0.05 | 0.27 | 1.23 | 8.12 | **90.32** | 1.94 |
| Grade ≥3 adverse events | | | | | | | |  |
| Alectinib | **90.30** | 7.46 | 1.50 | 0.54 | 0.17 | 0.03 | 0.00 | 97.85 |
| Brigatinib | 2.59 | 8.61 | 12.96 | 19.98 | 27.80 | 18.05 | 10.01 | 40.67 |
| Ceritinib | 0.31 | 1.21 | 1.92 | 6.91 | 18.00 | 39.04 | 32.61 | 18.56 |
| Crizotinib | 0.93 | 57.47 | 31.65 | 8.20 | 1.53 | 0.22 | 0.00 | 51.99 |
| Lorlatinib | 0.50 | 1.44 | 2.60 | 5.52 | 11.54 | 26.91 | **51.50** | 14.52 |
| Ensartinib | 4.93 | 16.91 | 20.87 | 21.34 | 19.12 | 11.25 | 5.59 | 51.85 |
| Chemotherapy | 0.44 | 6.92 | 28.50 | 37.51 | 21.85 | 4.50 | 0.28 | 18.56 |
| AE leading to discontinuation | | | | | | | | |
| Alectinib | 7.44 | 33.53 | 41.39 | 11.71 | 3.81 | 1.65 | 0.47 | 70.38 |
| Brigatinib | 1.33 | 2.89 | 4.78 | 9.93 | 12.19 | 24.07 | **44.80** | 19.97 |
| Ceritinib | **85.19** | 8.52 | 3.06 | 1.43 | 0.92 | 0.58 | 0.29 | 95.45 |
| Crizotinib | 0.06 | 0.60 | 4.13 | 35.68 | 40.30 | 16.73 | 2.50 | 37.37 |
| Lorlatinib | 1.65 | 3.55 | 6.07 | 14.93 | 23.69 | 30.04 | 20.08 | 29.02 |
| Ensartinib | 2.48 | 5.77 | 7.85 | 14.70 | 14.14 | 24.13 | 30.93 | 28.61 |
| Chemotherapy | 1.84 | 45.15 | 32.72 | 11.61 | 4.96 | 2.79 | 0.93 | 69.20 |

The number in each cell represents the posterior probability of the row-defining treatment being ranked at the columndefining position. The numbers with biggest probability of ranking first and last are in bold and underscored.

**Table S2: Comparisons of the fit of consistency and inconsistency models using deviance information criteria (DIC).**

| Model | PFS | OS | ORR | PFS-BM | Grade ≥3 AE | AE leading to discontinuation |
| --- | --- | --- | --- | --- | --- | --- |
| Consistency | 18.07 | 20.74 | 46.74 | 21.56 | 46.71 | 39.95 |
| Inconsistency | 17.62 | 21.68 | 46.88 | 22.87 | 47.44 | 40.68 |

The DIC is a Bayesian model evaluation criterion that measures model fit adjusted with complexity of the model; smaller DIC values correspond to more preferable models. PFS: Progression-free survival; OS: Overallsurvival; ORR: Objective response rate; PFS-BM: Progression-free Survival with brain metastases; AE: Adverse event.

**Table S3: Pooled estimates of the second sensitivity analysis stratifying patients by excluded the PROFILE 1029 study.**

| A | **Progression free survival(PFS)** | | | | | | | | | | | | | | | | | | | | | |
| --- | --- | --- | --- | --- | --- | --- | --- | --- | --- | --- | --- | --- | --- | --- | --- | --- | --- | --- | --- | --- | --- | --- |
| **Overall survival(OS)** | **Crizotinib** | | **0.42**  **(0.32, 0.55)** | | | 1.05  (0.68, 1.55) | | | **0.48**  **(0.31, 0.76)** | | | | **0.27**  **(0.16, 0.44)** | | | | **0.5**  **(0.31, 0.82)** | | | **2**  **(1.48, 2.59)** | | |
|  | 1.56  (0.99, 2.55) | | **Alecltinib** | | | **2.51**  **(1.53, 3.9)** | | | 1.15  (0.68, 1.93) | | | | 0.64  (0.36, 1.12) | | | | 1.21  (0.69, 2.08) | | | **4.79**  **(3.28, 6.64)** | | |
|  | 1  (0.52, 2.17) | | 0.64  (0.3, 1.48) | | | **Certinib** | | | **0.46**  **(0.25, 0.87)** | | | | **0.26**  **(0.14, 0.49)** | | | | **0.48**  **(0.26, 0.93)** | | | **1.91**  **(1.4, 2.6)** | | |
|  | 1.87  (0.8, 4.31) | | 1.2  (0.45, 3.1) | | | 1.87  (0.58, 5.29) | | | **Brigatinib** | | | | 0.56  (0.29, 1.09) | | | | 1.05  (0.54, 2.03) | | | **4.17**  **(2.39, 6.97)** | | |
|  | 1.09  (0.49, 2.44) | | 0.7  (0.27, 1.75) | | | 1.08  (0.35, 3.03) | | | 0.58  (0.18, 1.86) | | | | **Lorlatinib** | | | | 1.88  (0.93, 3.77) | | | **7.46**  **(4.17, 12.98)** | | |
|  | 1.1  (0.48, 2.48) | | 0.7 (  0.26, 1.78) | | | 1.09  (0.35, 3.08) | | | 0.59  (0.18, 1.91) | | | | 1.01  (0.32, 3.17) | | | | **Ensartinib** | | | **3.96**  **(2.23, 6.83)** | | |
|  | 0.85  (0.57, 1.46) | | 0.55  (0.31, 1.03) | | | 0.85  (0.5, 1.47) | | | 0.46  (0.18, 1.27) | | | | 0.79  (0.33, 2.09) | | | | 0.78  (0.32, 2.14) | | | **Chemotherapy** | | |
| B | **Objective response rate(ORR)** | | | | | | | | | | | | | | | | | | | |  | |
| PFS with brain metastases | **Crizotinib** | | | | 1.56  (0.94, 2.78) | | | 1.41  (0.51, 3.71) | | 1.4  (0.53, 3.76) | | 1.68  (0.63, 4.57) | | | 1.23  (0.46, 3.3) | | | **0.3**  **(0.14, 0.53)** | | |  | |
|  | **4.46**  **(2.33, 11.28)** | | | | **Alecltinib** | | | 0.91  (0.29, 2.5) | | 0.9  (0.28, 2.68) | | 1.08  (0.34, 3.25) | | | 0.79  (0.25, 2.32) | | | **0.19**  **(0.08, 0.37)** | | |  | |
|  | 0.88  (0.27, 2.97) | | | | **0.2**  **(0.05, 0.64)** | | | **Ceritinib** | | 0.99  (0.25, 4.09) | | 1.19  (0.3, 4.98) | | | 0.87  (0.23, 3.61) | | | **0.21**  **(0.09, 0.42)** | | |  | |
|  | 3.39  (0.92, 12.73) | | | | 0.76  (0.14, 3.01) | | | 3.87  (0.65,22.93) | | **Brigatinib** | | 1.21  (0.3, 4.82) | | | 0.88  (0.22, 3.56) | | | **0.21**  **(0.06, 0.64)** | | |  | |
|  | **4.81**  **(1.2, 19.12)** | | | | 1.08  (0.19, 4.61) | | | 5.47  (0.86,33.28) | | 1.42  (0.21, 9.39) | | **Lorlatinib** | | | 0.73  (0.18, 2.95) | | | **0.18**  **(0.05, 0.53)** | | |  | |
|  | 1.82  (0.49, 6.88) | | | | 0.41  (0.07, 1.66) | | | 2.07  (0.33,12.28) | | 0.53  (0.08, 3.45) | | 0.38  (0.06, 2.59) | | | **Ensartinib** | | | **0.24**  **(0.07, 0.71)** | | |  | |
|  | 0.53  (0.25, 1.22) | | | | **0.12**  **(0.04, 0.27)** | | | 0.61  (0.25, 1.5) | | **0.16**  **(0.03, 0.75)** | | **0.11**  **(0.02, 0.56)** | | | 0.29  (0.06, 1.42) | | | **Chemotherapy** | | |  | |
| C | | **Adverse events of grade 3 or higher** | | | | | | | | | | | | | | | | | | | |  |
| **AE leading to discontinuation** | | **Crizotinib** | | **0.64**  **(0.44, 0.88)** | | | 1.61  (0.89, 2.83) | | | | 1.45  (0.79, 2.66) | | | **2.15**  **(1.17, 3.99)** | | 1.26  (0.68, 2.36) | | | 1.05  (0.7, 1.54) | | |  |
|  |  | 1.59  (0.9, 2.94) | | **Alecltinib** | | | **2.52**  **(1.36, 4.82)** | | | | **2.28**  **(1.17, 4.71)** | | | **3.37 (1.73, 7.06)** | | 1.98  (1, 4.17) | | | **1.65**  **(1.04, 2.7)** | | |  |
|  |  | 3.07  (0.88, 9.96) | | 1.93  (0.51, 6.61) | | | **Certinib** | | | | 0.9  (0.4, 2.1) | | | 1.34  (0.59, 3.16) | | 0.78  (0.34, 1.86) | | | 0.65  (0.43, 1) | | |  |
|  |  | 0.69  (0.24, 1.96) | | 0.44  (0.13, 1.39) | | | 0.23  (0.05, 1.16) | | | | **Brigatinib** | | | 1.48  (0.63, 3.5) | | 0.87  (0.36, 2.06) | | | 0.72  (0.35, 1.48) | | |  |
|  |  | 0.86  (0.36, 2.03) | | 0.54  (0.19, 1.49) | | | 0.28  (0.07, 1.29) | | | | 1.25  (0.32, 4.89) | | | **Lorlatinib** | | 0.58  (0.24, 1.41) | | | 0.49  (0.23, 1) | | |  |
|  |  | 0.81  (0.26, 2.46) | | 0.51  (0.14, 1.76) | | | 0.26  (0.05, 1.43) | | | | 1.18  (0.25, 5.47) | | | 0.94  (0.23, 3.85) | | **Ensartinib** | | | 0.84  (0.4, 1.72) | | |  |
|  |  | 0.75  (0.13, 4.54) | | 0.51  (0.09, 2.69) | | | 0.46  (0.19, 1.08) | | | | 2.04  (0.51, 7.62) | | | 1.63  (0.47, 5.16) | | 1.72  (0.41, 6.81) | | | **Chemotherapy** | | |  |

1. Pooled hazard ratios (95% credible intervals) for progression-free survival (upper triangle) and overall survival (lower triangle). (B) Pooled odds ratios (95% credible intervals) for objective response rate (upper triangle) and progression-free survival with brain metastases(BM)(lower triangle). (C) Pooled hazard ratios (95% credible intervals) for grade ≥3 adverse events(upper triangle) and adverse events leading to treatment discontinuation(lower triangle). Significant results are in bold, and difference with the original analysis are in underlined.

**Table S4: SUCRA results of the second sensitivity analysis stratifying patients by excluded the PROFILE 1029 study.**

| Treatment | PFS | | | OS | ORR | | | PFS-BM | ADR Grade≥3 | | AE leading to discontinuation | |  |
| --- | --- | --- | --- | --- | --- | --- | --- | --- | --- | --- | --- | --- | --- |
| 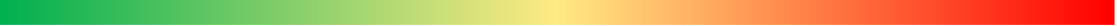 |  | | |  |  | | |  |  | |  | |  |
| Alectinib | | 75.61% | 79.96% | | | 71.57% | 84.23% | | | 98.80% | | 73.65% | |
| Brigatinib | | 64.42% | 86.24% | | | 60.85% | 72.43% | | | 34.40% | | 20.45% | |
| Certinib | | 23.81% | 38.83% | | | 61.47% | 25.51% | | | 25.00% | | 94.11% | |
| Chemotherapy | | 0.13% | 16.90% | | | 0.52% | 3.53% | | | 65.44% | | 61.81% | |
| Crizotinib | | 26.98% | 36.29% | | | 29.93% | 29.27% | | | 71.62% | | 39.93% | |
| Ensartinib | | 61.16% | 46.24% | | | 50.92% | 50.93% | | | 46.95% | | 29.81% | |
| Lorlatinib | | 97.90% | 45.54% | | | 74.74% | 84.11% | | | 7.79% | | 30.22% | |

Surface under the cumulative ranking curve (SUCRA) results of OS, ORR and adverse events of the second sensitivity analysis stratifying patients by excluded the PROFILE 1029 study.

**Table S5: The Egger’s and Begg’s test of the primary endpoints and adverse events.**

| Effect | Egger’s test | | Begg’s test | |
| --- | --- | --- | --- | --- |
|  | t | P | Z | P |
| PFS | -2.36 | **0.0397** | -2.13 | **0.0335** |
| OS | -1.28 | 0.2283 | -1.17 | 0.2437 |
| ORR | 0.69 | 0.5083 | -0.34 | 0.7317 |
| PFS-BM | -3.46 | **0.0061** | -2.47 | **0.0133** |
| AE grade≥3 | 0.02 | 0.985 | -0.07 | 0.94533 |
| AE leading to treatment discontinuation | -0.37 | 0.7196 | 0 | 1.000 |

(A) PFS: Progression-free survival; (B) OS: Overallsurvival; (C) ORR: Objective response rate; (D) PFS-BM: Progression-free Survival with brain metastases; (E) Adverse event grade ≥3; (F) Adverse event leading to treatment discontinuation. P value>0.5 means a potential publication bias

**References**

[1]Makoto Nishio, Dong-Wan Kim, Yi-Long Wu, et al., Crizotinib versus Chemotherapy in Asian Patients with ALK-Positive Advanced Non-small Cell Lung Cancer. Cancer Res Treat. 2018, 50(3):691-700.

[2]Benjamin J. Solomon, Dong-Wan Kim, Yi-Long Wu, et al., Final Overall Survival Analysis From a Study Comparing First-Line Crizotinib Versus Chemotherapy in ALK-Mutation-Positive Non-Small-Cell Lung Cancer. J Clin Oncol. 2018, 36(22): 2251-2258.

[3]Yi-Long Wu, Shun Lu, You Lu, et al., Results of PROFILE1029, a Phase III Comparison of First-Line Crizotinib versus Chemotherapy in East Asian Patients with ALK-Positive Advanced Non–Small Cell Lung Cancer, Journal of Thoracic Oncology, 13(10): 1539-1548.

[4]Alice T Shaw, Todd M Bauer, Filippo de Marinis, et al., First-Line Lorlatinib or Crizotinib in Advanced ALK-Positive Lung Cancer. Clinical Trial N Engl J Med. 2020, 383(21):2018-2029.

[5]Leora Horn, Ziping Wang, Gang Wu, et al., Ensartinib vs Crizotinib for Patients With Anaplastic Lymphoma Kinase-Positive Non–Small Cell Lung Cancer A Randomized Clinical Trial, JAMA Oncol. 2021,7(11):1617-1625.

[6]Kazuhiko Nakagawaa, Toyoaki Hidab, Hiroshi Nokihara, et al., Final progression-free survival results from the J-ALEX study of alectinib versus crizotinib in ALK-positive non-small-cell lung cancer, Lung Cancer, 2020, 139: 195-199.

[7]T. Mok1, Camidge, Gadgeel, et al., Updated overall survival and final progression-free survival data for patients with treatment-naive advanced ALK-positive non-small-cell lung cancer in the ALEX study. Annals of Oncology. 2020, 31(8): 1056-1064.

[8]Alice T Shaw, Tae Min Kim, Lucio Crinò, et al., Ceritinib versus chemotherapy in patients with ALK-rearranged non-small-cell lung cancer previously given chemotherapy and crizotinib (ASCEND-5): a randomised, controlled, open-label, phase 3 trial. Lancet Oncol. 2017, 18(7): 874-886.

[9]Jean-Charles Soria, Daniel S W Tan, Rita Chiari, et al., First-line ceritinib versus platinum-based chemotherapy in advanced ALK-rearranged non-small-cell lung cancer (ASCEND-4): a randomised, open-label, phase 3 study, Lancet, 2017, 389(10072):917-929.
[10]D. Ross Camidge, Hye Ryun Kim, Myung-Ju Ahn, et al. Brigatinib Versus Crizotinib in ALK Inhibitor-Naive Advanced ALK-Positive NSCLC: Final Results of Phase 3 ALTA-1L Trial. Journal of Thoracic Oncology, 2021, 16(12): 2091-2108.

[11]Novello S, Mazières J, Oh IJ, et al., Alectinib versus chemotherapy in crizotinibpretreated anaplastic lymphoma kinase (ALK)-positive non-small-cell lung cancer: results from the phase III ALUR study. Ann Oncol. 2018, 29(6):1409-1416.

[12]Caicun Zhou, Sang-We Kim, Thanyanan Reungwetwattana, et al., Alectinib versus crizotinib in untreated Asian patients with anaplastic lymphoma kinase-positive non-small-cell lung cancer (ALESIA): a randomised phase 3 study. Lancet Respir Med. 2019, 7(5): 437-446.
